# SARS-CoV-2 self-test uptake and factors associated with self-testing during Omicron BA.1 and BA.2 waves in France, January to May 2022

**DOI:** 10.2807/1560-7917.ES.2023.28.18.2200781

**Published:** 2023-05-04

**Authors:** Olivier Supplisson, Tiffany Charmet, Simon Galmiche, Laura Schaeffer, Olivia Chény, Anne Lévy, Nathan Jeandet, Faïza Omar, Christophe David, Alexandra Mailles, Arnaud Fontanet

**Affiliations:** 1 Institut Pasteur, Université Paris Cité, Emerging Diseases Epidemiology Unit, Paris, France; 2 Center for Interdisciplinary Research in Biology, Ecology and Evolution of Health team (Collège de France, CNRS/UMR 7241, Inserm U1050), Paris, France; 3 Sorbonne Université, Paris, France; 4 Institut Pasteur, Université Paris Cité, Clinical Operation Coordination Office, Paris, France; 5 Caisse Nationale d’Assurance Maladie, Paris, France; 6 Institut IPSOS, Paris, France; 7 Santé Publique France, Saint-Maurice, France; 8 Conservatoire National des Arts et Métiers, unité PACRI, Paris, France

**Keywords:** SARS-CoV-2, Omicron, Self-tests, At-home tests, Self-test uptake, Testing behavior

## Abstract

**Background:**

Following the SARS-CoV-2 Omicron variant spread, the use of unsupervised antigenic rapid diagnostic tests (self-tests) increased.

**Aim:**

This study aimed to measure self-test uptake and factors associated with self-testing.

**Methods:**

In this cross-sectional study from 20 January to 2 May 2022, the case series from a case–control study on factors associated with SARS-CoV-2 infection were used to analyse self-testing habits in France. A multivariable quasi-Poisson regression was used to explore the variables associated with self-testing among symptomatic cases who were not contacts of another infected individual. The control series from the same study was used as a proxy for the self-test background rate in the non-infected population of France.

**Results:**

During the study period, 179,165 cases who tested positive through supervised tests were recruited. Of these, 64.7% had performed a self-test in the 3 days preceding this supervised test, of which 79,038 (68.2%) were positive. The most frequently reported reason for self-testing was the presence of symptoms (64.6%). Among symptomatic cases who were not aware of being contacts of another case, self-testing was positively associated with being female, higher education, household size, being a teacher and negatively associated with older age, not French by birth, healthcare-related work and immunosuppression. Among the control series, 12% self-tested during the 8 days preceding questionnaire filling, with temporal heterogeneity.

**Conclusion:**

The analysis showed high self-test uptake in France with some inequalities which must be addressed through education and facilitated access (cost and availability) for making it a more efficient epidemic control tool.

**Key public health message**

**What did you want to address in this study?**

Self-tests carry the promise to make people more autonomous in their COVID-19 disease management. We wished to investigate the use of self-tests and the characteristics (sociodemographic and medical) associated with their uptake in France during the SARS-CoV-2 Omicron-BA.1 and BA.2 epidemic waves (from 20 January to 2 May 2022).

**What have we learnt from this study?**

The French population quickly and widely adopted self-testing, with 0.5 to 2 million people self-testing every day during the study period (BA.1 and BA.2 omicron waves). Use of self-test was motivated primarily by the presence of symptoms, or a history of contact with an infected person. It was however less common in older people, those with diploma lower than high school level and those not French by birth.

**What are the implications of your findings for public health?**

The analysis reveals inequalities in SARS-CoV-2 testing uptake, which must be addressed through easier access and better communication for self-testing to be used as a more efficient control tool during future epidemic waves.

## Introduction

The worldwide upscale of testing capabilities during the COVID-19 pandemic was accompanied by scientific contributions aiming to optimise the transition from pandemic response to pandemic control [1,2]. There are now two types of tests available for COVID-19 diagnosis: quantitative reverse transcription PCR (RT-qPCR), relying on molecular testing and antigen rapid-diagnostic tests (Ag-RDTs), relying on the detection of severe acute respiratory syndrome (SARS-CoV-2) viral proteins. The more expensive RT-qPCR tests have a longer turnaround time compared with Ag-RDTs, but they have a lower limit of detection, which makes RT-qPCR tests capable of detecting infected individuals before or in the early part of the infectious period [3], whereas Ag-RDTs can typically only detect viral proteins after the individual has become infectious [4-8]. The Ag-RDTs can either be performed under the supervision of a healthcare provider or by individuals themselves, using self-tests. In addition to being more convenient for individuals, self-tests are significantly cheaper than supervised Ag-RDTs. From a public health perspective, self-tests may be a stepping stone for successfully managing the pandemic-to-endemic phase by decreasing turnaround time, ease of use, compliance, costs for society and enabling specific testing regimens [4,6,9-11].

Although a formal evaluation of self-tests and how they compared with supervised Ag-RDTs was still lacking [12-16], in late April 2021 the French National Authority for Health (Haute Autorité de Santé (HAS)) ruled in favour of using self-tests for asymptomatic individuals during large-scale targeted iterative screening (instead of supervised Ag-RDTs), or for asymptomatic individuals for private use. Following the SARS-CoV-2 Omicron variant (Phylogenetic Assignment of Named Global Outbreak (Pango) lineage designation B.1.1.529) subvariant BA.1 surge in late December 2021, HAS extended its recommendations to self-test to individuals with a complete vaccination scheme who had been in contact with a known SARS-CoV-2 infected individual [16]. These guidelines also recommended confirming any positive self-test with a supervised test. Despite increasing availability and recommendations, self-test uptake levels and the associated factors remained poorly known. Identifying factors associated with low self-test uptake could help design a targeted public health policy for populations less likely to self-test. The aim of our study was therefore to measure self-test uptake and to identify which factors are associated with low self-test uptake.

## Methods

### Study design and setting

Details regarding the case–control study (ComCor study) have been previously described [17-19]. In brief, the ComCor study was a French nationwide case–control study, whose purpose was to identify factors associated with SARS-CoV-2 infection.

Cases were identified using SARS-CoV-2 infection diagnoses, either through RT-qPCR or laboratory or pharmacy-based supervised Ag-RDTs (thereafter named index tests). These positive results were obtained through the national monitoring and testing database for COVID-19 (SI-DEP), a nationwide routinely collected dataset powered by the French national health insurance (CNAM). It contained information on all French adults with a SARS-CoV-2 infection identified through a supervised test during the previous week. All adults with a recent SARS-CoV-2 infection diagnosis through supervised test between 1 October 2020 and 31 August 2022 and who had previously provided their email address to the CNAM (55% of all people with national health insurance), were invited to participate in the ComCor study.

Controls, sampled from the French population, were provided by Ipsos, a market research and public opinion specialist company. Controls were adults with no documented recent SARS-CoV-2 infection (no negative test required before inclusion), recruited to match (frequency-matching) cases based on age (18–29, 30–54 and ≥55 years), sex, region (the largest administrative area in France, with 13 regions for the country), population size (less than 5,000 inhabitants; 5,000–19,999 inhabitants; 20,000–99,999 inhabitants; 100,000 + inhabitants; and Paris urban area) and calendar week. Inclusion criteria for controls were adults invited by Ipsos through email who agreed to participate. Exclusion criteria for cases and controls were legal protection measures such as guardianship or conservatorship.

Questions regarding self-testing habits were added to the questionnaire on 20 January 2022 for both cases and controls. Cases were asked whether they performed a self-test in the preceding 3 days of the index test. Controls were asked to report any self-test uptake during the 8 days preceding their questionnaire filling. Participants who reported performing at least one self-test were asked the reason for their self-test uptake, and for cases, the reason for seeking a confirmatory supervised test after their self-test. The sanitary pass was a document required for frequentation of various areas such as restaurants, movie theatres, museums or healthcare facilities. This requirement started on 6 June 2021 and ended on 14 March 2022, when it became required only in healthcare facilities. The sanitary pass required either a recent (> 11 days and < 4 months) positive supervised test, a vaccination certificate or a recent (< 24 hours) negative supervised test result.

Supplementary Figure S1 presents the key dates related to the study.

### Population selection

The study population was restricted to participants from metropolitan France who completed the questionnaire between 20 January 2022 (date at which the questions on self-testing were introduced) and 2 May 2022 (date of study closure for this analysis). For cases, the study population was further restricted to those who had symptoms or index tests after 3 January 2022, to ensure that self-tests were readily accessible to individuals who wanted to use them. However, between 20 January and 15 February 2020, weekly invites were capped to randomly selected 400,000 cases to account for online questionnaire server capacity. Questionnaires showing inconsistencies in dates of self-tests and confirmatory tests were excluded.

### Statistical analysis

In this analysis, cases were used to estimate self-test uptake among SARS-CoV-2 infected individuals and factors associated with self-test uptake among symptomatic cases. Controls were used to estimate self-test uptake among non-infected individuals as a proxy for the self-test background rate in the non-infected population in France. Results for controls are shown at the end of the Results section and as Supplementary material.

To identify factors associated with self-testing uptake among people experiencing symptoms related to COVID-19, we performed a multivariable analysis among cases who were symptomatic when they performed their supervised test and tested because of other reasons than contact with an infected individual. The dependent variable took the value 1 when at least one self-test was performed in the presence of symptoms during the 3 days preceding the index test, and 0 otherwise. Characteristics associated with self-test uptake were analysed through risk ratio (RR) estimates obtained using a modified Poisson approach, a quasi-maximum likelihood estimator approach (QMLE, see [20,21]). To use such an estimator, the dependent variable does not need to follow a Poisson distribution [20,22]. It is asymptotically Gaussian [20,21] and provides consistent estimates of the conditional mean parameters, even under overdispersion [20,22,23]). Given our very large sample size, asymptotic properties of QMLE estimators were assumed to hold.

Because of the risk of post-selection inference bias, we did not use data-driven variable selection procedures [24]. Risk ratios (RR) were obtained for both the univariable (unadjusted) and multivariable (adjusted) analysis. Multivariable analysis was fitted using all variables displayed in Tables 1, 2 and 3 such as socio-demographic, exposure and health-related characteristics, and included week of symptom onset in the single regression model. The only variable that was collected as continuous was age, from which we created six categories based on the cut-offs of previous ComCor analyses. All other variables were qualitative and all of them had also been included in past ComCor articles. Pairwise correlations were low, excluding any multicollinearity issue (see Supplementary Figure S2).

**Table 1:** Sociodemographic characteristics associated with self-test uptake among symptomatic SARS-CoV-2 cases, France, January–May 2022

Variables	Recruited cases^a^		Symptomatic recruited cases who tested for other reasons than contact with another SARS-CoV-2 infected individual^b^		Self-test uptakers^c^		Univariable analysis		Multivariable analysis	
	n (N = 179,165)	Column % ^d^	n (N = 75,463)	Column % ^d^	n (N = 44,132)	Row % ^e^	RR	95% CI	RR	95% CI
**Sex**										
Male	50,095	28.0	22,170	29.4	12,467	56.2	Ref			
Female	129,070	72.0	53,293	70.6	31,665	59.4	1.06	1.04–1.07	1.03	1.01–1.04
**Age (years)**										
18–29	16,795	9.4	7,479	9.9	4,061	54.3	Ref			
30–39	41,292	23.0	15,434	20.5	9,598	62.2	1.15	1.12–1.17	1.02	1–1.05
40–49	49,761	27.8	18,669	24.7	12,051	64.6	1.19	1.16–1.22	1.02	1–1.05
50–59	37,773	21.1	17,992	23.8	10,503	58.4	1.08	1.05–1.1	1.00	0.97–1.03
60–69	22,516	12.6	10,624	14.1	5,517	51.9	0.96	0.93–0.98	0.93	0.9–0.97
≥70	11,028	6.2	5,265	7.0	2,402	45.6	0.84	0.81–0.87	0.83	0.79–0.88
**Level of education**										
Lower than high school level	30,437	17.0	13,401	17.8	6,915	51.6	Ref			
High school level	33,877	18.9	14,201	18.8	8,079	56.9	1.10	1.08–1.13	1.07	1.05–1.09
Bachelor's degree level	68,622	38.3	28,201	37.4	17,335	61.5	1.19	1.17–1.21	1.12	1.10–1.15
Master's degree level or higher	46,229	25.8	19,660	26.1	11,803	60.0	1.16	1.14–1.19	1.14	1.11–1.16
**French citizenship**										
Yes, by birth	166,553	93	69,849	92.6	41,311	59.1	Ref			
No	5,336	3	2,426	3.2	1,219	50.2	0.85	0.82–0.88	0.90	0.87–0.94
Yes, by naturalisation, marriage, etc.	6,326	3.5	2,750	3.6	1,387	50.4	0.85	0.82–0.89	0.89	0.86–0.93
**Professional categories**										
Worker	7,401	4.1	3,207	4.2	1,753	54.7	Ref			
Independent profession (including farmers)	4,956	2.8	2,205	2.9	1,313	59.5	1.09	1.04–1.14	1.07	1.02–1.12
Employee	35,232	19.7	14,482	19.2	8,527	58.9	1.08	1.04–1.11	1.05	1.01–1.09
Intermediate profession	40,067	22.4	16,081	21.3	9,939	61.8	1.13	1.09–1.17	1.07	1.03–1.11
Senior executive	53,720	30.0	22,704	30.1	14,124	62.2	1.14	1.10–1.18	1.08	1.04–1.12
Unemployed or inactive people^f^	11,452	6.4	4,508	6.0	2,402	53.3	0.97	0.93–1.02	1.01	0.97–1.06
Retired	26,337	14.7	12,276	16.3	6,074	49.5	0.91	0.87–0.94	1.08	1.03–1.14
**Population size of place of residence**										
Less than 5,000 inhabitants	49,960	27.9	19,316	25.6	12,254	63.4	Ref			
5,000–19,999 inhabitants	19,783	11.0	8,082	10.7	5,071	62.7	0.99	0.97–1.01	1 .00	0.98–1.02
20,000–99,999 inhabitants	22,769	12.7	9,625	12.8	5,827	60.5	0.95	0.94–0.97	1.00	0.98–1.02
≥100,000 inhabitants (including Paris urban area)	86,653	48.4	38,440	50.9	20,980	54.6	0.86	0.85–0.87	0.95	0.93–0.96
**Region**										
Île-de-France (Paris region)	32,875	18.4	15,003	19.9	7,937	52.9	Ref			
Centre - Val de Loire	6,588	3.7	2,724	3.6	1,771	65.0	1.23	1.19–1.27	1.12	1.09–1.16
Bourgogne -Franche-Comté	7,973	4.4	3,228	4.3	1,931	59.8	1.13	1.10–1.17	1.06	1.02–1.09
Normandie	8,758	4.9	3,478	4.6	2,162	62.2	1.18	1.14–1.21	1.08	1.04–1.11
Hauts-de-France	16,143	9.0	6,713	8.9	3,676	54.8	1.04	1.01–1.06	0.95	0.93–0.98
Grand Est	18,464	10.3	7,513	10.0	4,398	58.5	1.11	1.08–1.13	1.04	1.01–1.06
Pays de la Loire	9,601	5.4	3,991	5.3	2,645	66.3	1.25	1.22–1.29	1.15	1.12–1.18
Bretagne	10,042	5.6	3,931	5.2	2,531	64.4	1.22	1.18–1.25	1.11	1.08–1.14
Nouvelle-Aquitaine	17,395	9.7	6,706	8.9	4,220	62.9	1.19	1.16–1.22	1.11	1.08–1.14
Occitanie	17,355	9.7	7,279	9.6	4,300	59.1	1.12	1.09–1.14	1.05	1.02–1.07
Auvergne-Rhône-Alpes	20,811	11.6	9,028	12.0	5,506	61.0	1.15	1.13–1.18	1.1	1.07–1.12
Provence-Alpes-Côte d’Azur and Corse	13,160	7.3	5,869	7.8	3,055	52.0	0.98	0.96–1.01	0.97	0.95–1.00

CI: confidence interval; Ref: reference value; RR: risk ratio.

^a^ Individuals who tested positive through supervised test (either supervised Ag-RDT or RT-qPCR), who were invited to participate, who agreed to do so, and who were kept in the study population following the population selection process.

^b^ Multivariable analysis was restricted to participants who did not test because of contact with an infected individual and were symptomatic when they did both their supervised test and their self-test, for self-test uptakers, and their supervised test only for those who did not self-test. Variables with missing values are available in Supplementary Table S4. Multivariable analysis was fitted using all variables displayed in Tables 1, 2 and 3 - socio-demographic, exposure, and health-related characteristics - and week of symptom onset (Supplementary Table S5) in a single regression model.

^c^ Among recruited symptomatic cases who did not test because of contact with a SARS-CoV-2 infected individual.

^d^ Column %: proportion by variable category.

^e^ Row %: proportion of the second column (b) who performed a self-test according to the variable category.

^f^ Inactive people include students, househusbands and housewives and people who cannot work because of handicaps, in accordance with the French statistics institute (INSEE).

**Table 2:** Exposure-related variables associated with self-test uptake among symptomatic SARS-CoV-2 cases, France, January–May 2022

Variables	Recruited cases^a^		Symptomatic recruited cases who tested for other reasons than contact with another SARS-CoV-2 infected individual^b^		Self-test uptakers^c^		Univariable analysis		Multivariable analysis	
	n (N = 179,165)	Column % ^d^	n (N = 75,463)	Column % ^d^	n (N = 44,132)	Row % ^e^	RR	95% CI	RR	95% CI
**Healthcare worker**										
Not healthcare professional	158,192	88.3	66,918	88.7	39,421	58.9	Ref			
Administrative/management staff	2,754	1.5	1,197	1.6	642	53.6	0.91	0.86–0.96	0.86	0.82–0.91
Assistant nurse	2,640	1.5	958	1.3	520	54.3	0.92	0.87–0.98	0.87	0.82–0.93
Nurse	4,924	2.8	2,000	2.7	1,153	57.6	0.98	0.94–1.02	0.87	0.83–0.90
General and specialist physician	1,414	0.8	630	0.8	310	49.2	0.84	0.77–0.90	0.77	0.71–0.84
Pharmacist	1,226	0.7	534	0.7	292	54.7	0.93	0.86–1.00	0.85	0.79–0.92
Other	8,015	4.5	3,226	4.3	1,794	55.6	0.94	0.91–0.97	0.89	0.86–0.92
**Location of work-related activity**										
Office work with no remote working	33,392	18.6	15,074	20.0	9,163	60.8	Ref			
Not working	36,967	20.6	16,240	21.5	8,230	50.7	0.83	0.82–0.85	0.96	0.93–0.99
Working but no office work	52,310	29.2	22,051	29.2	13,497	61.2	1.01	0.99–1.02	1.00	0.99–1.02
Split office/ remote working	28,798	16.1	12,189	16.2	7,404	60.7	1.00	0.98–1.02	1.00	0.98–1.02
Complete remote working	9,612	5.4	2,930	3.9	1,709	58.3	0.96	0.93–0.99	0.97	0.94–1.00
On holiday during the whole period	18,085	10.1	6,979	9.2	4,129	59.2	0.97	0.95–1.00	0.98	0.96–1.01
**Teaching-related activities**										
No teaching	168,643	94.1	71,136	94.3	40,725	57.2	Ref			
Teacher at kindergarten	2,100	1.2	764	1.0	658	86.1	1.50	1.46–1.55	1.28	1.24–1.33
Teacher at primary school	1,494	0.8	553	0.7	464	83.9	1.47	1.41–1.52	1.24	1.19–1.30
Teacher at middle school	1,892	1.1	808	1.1	675	83.5	1.46	1.41–1.51	1.25	1.20–1.30
Teacher at high school	1,597	0.9	722	1.0	584	80.9	1.41	1.36–1.46	1.22	1.17–1.28
Teacher at college/university	1,036	0.6	462	0.6	305	66.0	1.15	1.08–1.23	1.08	1.01–1.16
Teacher in continuous education service	507	0.3	214	0.3	131	61.2	1.07	0.96–1.19	0.97	0.87–1.08
Teacher at art institution	228	0.1	103	0.1	65	63.1	1.10	0.95–1.28	1.08	0.94–1.25
Teacher, other	439	0.2	178	0.2	111	62.4	1.09	0.97–1.22	1.01	0.90–1.12
Teacher at multiple school levels	1,229	0.7	523	0.7	414	79.2	1.38	1.32–1.45	1.23	1.17–1.29
**Child in household**										
No children	88,905	49.6	43,808	58.1	23,145	52.8	Ref			
Child attending daycare centre	2,694	1.5	1,024	1.4	592	57.8	1.09	1.04–1.15	1.00	0.94–1.06
Child looked after by a childminder	3,249	1.8	1,153	1.5	742	64.3	1.22	1.17–1.27	1.07	1.02–1.12
Child attending kindergarten	6,171	3.4	2,123	2.8	1,404	66.1	1.25	1.21–1.29	1.13	1.09–1.17
Child attending primary school	11,554	6.4	3,597	4.8	2,505	69.6	1.32	1.29–1.35	1.17	1.14–1.20
Child attending middle school	8,152	4.6	3,128	4.1	2,020	64.6	1.22	1.19–1.26	1.09	1.05–1.12
Child attending high school	8,420	4.7	3,549	4.7	2,252	63.5	1.20	1.17–1.23	1.07	1.03–1.10
Child attending college or university	7,576	4.2	3,475	4.6	2,046	58.9	1.11	1.08–1.15	1.02	0.98–1.05
Multiple children attending several school levels	42,444	23.7	13,606	18.0	9,426	69.3	1.31	1.29–1.33	1.14	1.11–1.17
**Attending lectures in person**										
No	164,825	92.0	69,325	91.9	39,882	57.5	Ref			
Yes	14,340	8.0	6,138	8.1	4,250	69.2	1.2	1.18–1.23	1.05	1.02–1.08
**Housing type**										
House	117,899	65.8	46,827	62.1	29,506	63.0	Ref			
Apartment	60,819	34.0	28,441	37.7	14,554	51.2	0.81	0.80–0.82	0.89	0.88–0.91
Shelter or nursing home	447	0.2	195	0.3	72	36.9	0.59	0.49–0.70	0.70	0.59–0.84
**Household size**										
1	30,682	17.1	16,491	21.9	8,004	48.5	Ref			
2	56,683	31.6	26,714	35.4	14,981	56.1	1.16	1.13–1.18	1.11	1.09–1.13
3	35,867	20.0	14,115	18.7	8,818	62.5	1.29	1.26–1.31	1.11	1.08–1.14
4	40,046	22.4	13,276	17.6	9,155	69.0	1.42	1.39–1.45	1.14	1.10–1.17
5	12,292	6.9	3,724	4.9	2,481	66.6	1.37	1.34–1.41	1.10	1.06–1.14
6 +	3,595	2.0	1,143	1.5	693	60.6	1.25	1.19–1.31	1.07	1.01–1.13

CI: confidence interval; Ref: reference value; RR: risk ratio.

^a^ Individuals who tested positive through supervised test (either supervised Ag-RDT or RT-qPCR), who were invited to participate, who agreed to do so, and who were kept in the study population following the population selection process.

^b^ Multivariable analysis was restricted to participants who did not test because of contact with an infected individual and were symptomatic when they did both their supervised test and their self-test, for self-test uptakers, and their supervised test only for those who did not self-test. Variables with missing values are available in Supplementary Table S4. Multivariable analysis was fitted using all variables displayed in Tables 1, 2 and 3 - socio-demographic, exposure and health-related characteristics - and week of symptom onset (Supplementary Table S5) in a single regression model.

^c^ Among recruited symptomatic cases who did not test because of contact with a SARS-CoV-2 infected individual.

^d^ Column %: proportion by variable category.

^e^ Row %: proportion of the second column (b) who performed a self-test according to the variable category.

**Table 3:** Health-related variables associated with self-test uptake among symptomatic SARS-CoV-2 cases, France, January–May 2022

Variables	Recruited cases^a^		Symptomatic recruited cases who tested for other reasons than contact with another SARS-CoV-2 infected individual^b^		Self-test uptakers^c^		Univariable		Multivariable	
	n (N = 179,165)	Column % ^d^	n (N = 75,463)	Column %^d^	n (N = 44,132)	Row %^e^	RR	95% CI	RR	95% CI
**COVID-19 vaccination status**										
Unvaccinated, no history of past infection	10,213	5.7	4,433	5.9	2,641	59.6	Ref			
Unvaccinated, history of past infection	1,142	0.6	471	0.6	284	60.3	1.01	0.94–1.09	1.03	0.96–1.11
Incomplete primary vaccination series	506	0.3	211	0.3	116	55.0	0.94	0.84–1.05	0.95	0.85–1.06
Primary vaccination series < 3 months	12,401	6.9	4,814	6.4	2,867	59.6	1.00	0.96–1.03	0.96	0.93–1.00
Primary vaccination series 3–6 months	22,668	12.7	9,626	12.8	5,409	56.2	0.94	0.91–0.97	0.94	0.91–0.97
Primary vaccination series > 6 months	9,215	5.1	3,842	5.1	2,168	56.4	0.94	0.91–0.98	0.95	0.92–0.98
Booster < 3 months	63,554	35.5	24,093	31.9	14,757	61.2	1.03	1.00–1.06	0.98	0.96–1.01
Booster 3–6 months	45,059	25.1	21,475	28.5	12,397	57.7	0.96	0.94–0.99	0.99	0.96–1.02
Booster > 6 months	1,077	0.6	526	0.7	277	52.7	0.88	0.82–0.96	1.05	0.97–1.14
**Health consciousness**										
Not at all	959	0.5	338	0.4	179	53.0	Ref			
Yes, a little	13,657	7.6	5,381	7.1	3,225	59.9	1.13	1.02–1.25	1.09	0.99–1.21
Yes, rather	107,146	59.8	44,674	59.2	26,646	59.6	1.13	1.02–1.25	1.09	0.99–1.21
Yes, a lot	57,403	32.0	25,070	33.2	14,082	56.2	1.06	0.96–1.17	1.06	0.96–1.17
**Comorbidities**										
**Body-mass index**										
< 18.5	90,113	50.3	38,415	50.9	22,425	58.4	Ref			
18.5–24.9	5,377	3.0	2,202	2.9	1,240	56.3	0.96	0.93–1.00	0.98	0.94–1.01
25–30	53,513	29.9	22,586	29.9	13,225	58.5	1.00	0.99–1.02	1.02	1.00–1.03
> 30	30,162	16.8	12,260	16.2	7,242	59.1	1.01	0.99–1.03	1.02	1.01–1.04
**Immunosuppression**										
No	168,668	94.1	70,867	93.9	41,728	58.9	Ref			
Yes	8,684	4.8	3,829	5.1	1,990	52.0	0.88	0.86–0.91	0.94	0.91–0.97
**Hypertension**										
No	156,874	87.6	65,482	86.8	38,629	59.0	Ref			
Yes	19,817	11.1	8,989	11.9	4,930	54.8	0.93	0.91–0.95	1.02	1.00–1.04
**Coronary artery disease**										
No	174,753	97.5	73,587	97.5	30,510	41.46	Ref			
Yes	1,938	1.1	884	1.2	482	54.5	0.93	0.88–0.99	1.07	1.00–1.13
**Chronic respiratory diseases**										
No	160,927	89.8	68,135	90.3	39,945	58.6	Ref			
Yes	15,764	8.8	6,336	8.4	3,614	57.0	0.97	0.95–0.99	1.00	0.97–1.02
**Diabetes**										
No	170,843	93.87	71,958	95.4	42,256	58.7	Ref			
Yes	5,848	3.3	2,513	3.3	1,303	51.9	0.88	0.85–0.92	0.97	0.93–1.01

CI: confidence interval; Ref: reference value; RR: risk ratio.

^a^ Individuals who tested positive through supervised test (either supervised Ag-RDT or RT-qPCR), who were invited to participate, who agreed to do so and who were kept in the study population following the population selection process.

^b^ Multivariable analysis was restricted to participants who did not test because of contact with an infected individual and were symptomatic when they did both their supervised test and their self-test, for self-test uptakers, and their supervised test only for those who did not self-test. Variables with missing values are available in Supplementary Table S4. Multivariable analysis was fitted using all variables displayed in Tables 1, 2 and 3 - socio-demographic, exposure and health-related characteristics - and week of symptom onset (Supplementary Table S5) in a single regression model.

^c^ Among recruited symptomatic cases who did not test because of contact with a SARS-CoV-2 infected individual.

^d^ Column %: proportion by variable category.

^e^ Row %: proportion of the second column (b) who performed a self-test according to the variable category.

Missing data were not considered as specific categories in any of the regression to avoid biased estimates [25,26]. Instead, missing values were handled using multiple imputations by chained formula [26,27] using all variables included in the model and the outcome. For variables with missing values, the univariable RR estimated by the model was thus slightly different than it would have been if it had been manually computed. Estimates were pooled using Rubin’s method [28]. Analysis was performed using R software version 4.1.2 (R Foundation, Vienna, Austria) [29]. Multiple imputations were performed using the mice function, with five multiple imputations and five iterations from the mice package [30].

Sensitivity analysis was performed by considering a complete case approach, which is known to be unbiased when the missingness pattern does not depend on the targeted outcome [31,32]. Statistical significance was appreciated based on a type I error rate equal to 5%. Corresponding confidence intervals (CI) were constructed using the 0.975 normal distribution quantile and robust standard errors obtained using White’s estimator. In practice, robust standard errors were obtained using the lmtest [33] and sandwich [34,35] packages. Since this analysis was not planned at the initiation of the study, we did not calculate a sample size based on an expected increase in self-testing uptake associated with participants’ characteristics. The sample size happened to be the number of participants who responded to the questionnaire during the study period and who matched the criteria chosen for the analysis.

### Reporting guidelines

Consistency between the study report and the STROBE guidelines was assessed, see Supplementary Table S3.

## Results

### Self-test uptake among all recruited cases

**Figure 1:**
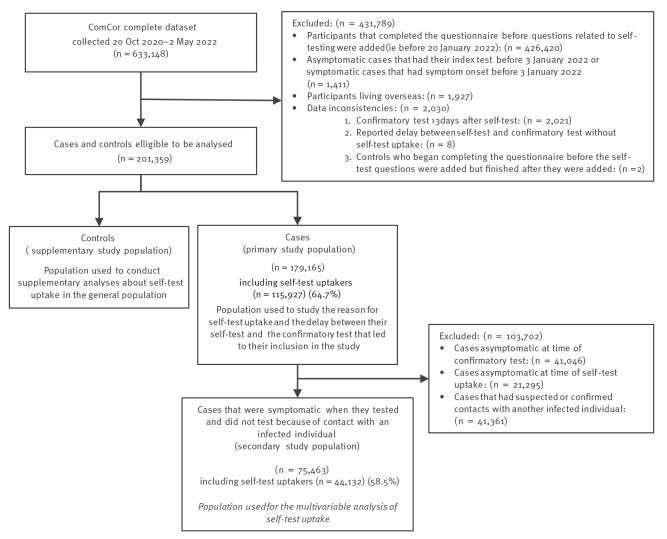
Study flowchart for the inclusion of SARS-CoV-2 cases and controls, France, January–May 2022

Between 20 January 2022 and 2 May 2022, 179,165 cases who tested positive with RT-qPCR or supervised Ag-RDT were recruited into the study population, see flowchart in Figure 1. A complete description of the characteristics of the population is available in columns 1 and 2 in Tables 1, 2 and 3.

**Figure 2:**
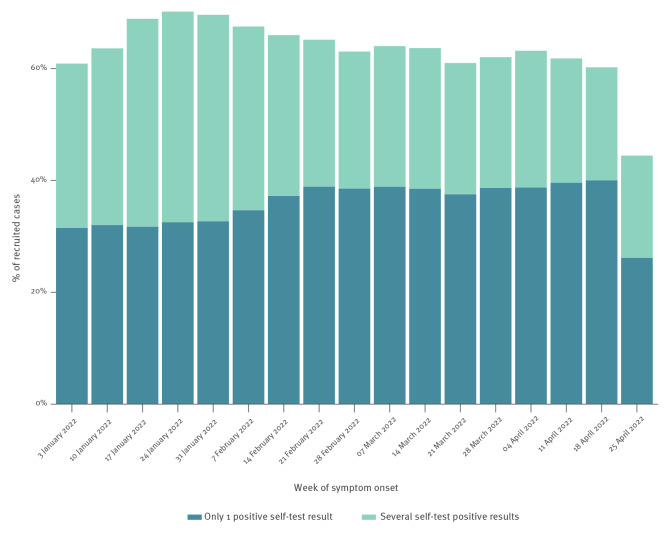
Self-test uptake prior to positive supervised tests among SARS-CoV-2 cases by week, France, January–May 2022

Among all the recruited cases, 72,528 (40.5%) declared a positive RT-qPCR and 121,292 (67.7%) declared a positive supervised Ag-RDT. For those who performed at least one self-test in the 3 days preceding the index test (n = 115,927, 64.7%), the self-test was positive for 79,038 (68.2%). Thus, 44.1% of recruited cases who tested positive through RT-qPCR or supervised Ag-RDT had a history of a recent (< 3 days) positive self-test at the time they went to a pharmacy or a laboratory for supervised testing. Self-test uptake among the study population by week of symptom onset was higher than 60% for almost all of the study period, with a peak at end of January at 70%, see Figure 2.

### Reasons for self-test uptake reported by all recruited cases

**Figure 3:**
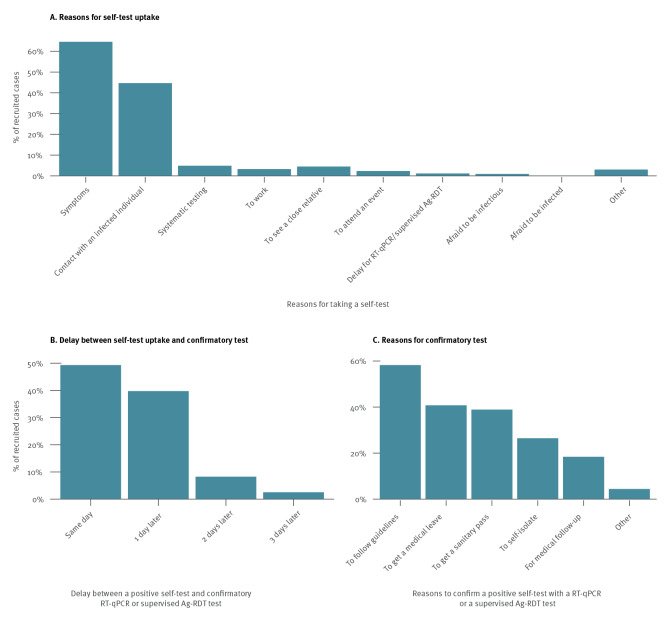
(A) Reasons for self-test uptake, (B) delay between self-test and confirmatory test and (C) reasons for confirmatory test among recruited symptomatic SARS-CoV-2 cases who took a self-test, France, January–May 2022

The primary reasons for self-testing among SARS-CoV-2 cases who tested positive through confirmatory supervised test and agreed to participate were symptoms (n = 74,926, 64.6%) and history of contact with an infected individual (n = 51,829, 44.7%). Among respondents with positive self-test, almost 90% confirmed the results with an RT-qPCR or a supervised Ag-RDT the same day or the next day. As exhibited in Figure 3C, participants decided to confirm the positive results mainly to follow the public health recommendations (n = 46,064, 58.3%), to obtain medical leave certificate (n = 32,237, 40.8%), or to receive a sanitary pass (n = 30,789, 39.0%). The evolution of reasons for self-test uptake is reported in Supplementary Figure S6.

### Factors associated with self-testing among recruited symptomatic cases who were not contacts

Sociodemographic, exposure-related and health-related characteristics associated with self-test uptake are described in Tables 1, 2 and 3, respectively. Details about missing values are provided in Supplementary Table S4 and results for the week of symptom onset are available in Supplementary Table S5.

Among symptomatic cases who tested positive through a supervised confirmatory test and self-tested because of reasons other than contact with an infected individual, self-testing was positively associated with being female, having a higher level of education and living in another region than Île-de-France or Hauts-de-France. It was negatively associated with being older than 60 years of age (individuals aged 60–69 or ≥70 years were significantly less likely to self-test than younger participants), not being a French national by birth and living in a densely populated city.

Regarding exposure-related characteristics, self-test uptake was significantly associated with the number of people living in the household, living or working (teacher) with a child and having lectures in person. It was negatively associated with being a healthcare worker, not working and living in an apartment or a shelter/nursing home.

Finally, we found that self-test uptake was less common among participants with immunosuppression. They also did not preferentially opt for RT-qPCR compared with Ag-RDT testing for diagnosis, so their lower use of self-testing was unlikely motivated by a preference for more sensitive (i.e. RT-qPCR) testing (see Supplementary Figure S7). Altogether, the magnitude of differences in self-testing among patients with or without comorbidities was small, and partially confounded by age so it is unlikely that health condition played a significant role in self-testing choices.

Point estimates remained stable when considering complete cases analysis, and 95% CIs showed minimal differences compared to the analysis by multiple imputations, see Supplementary Table S8.

### Supplementary analysis on recruited controls

Between 20 January and 2 May 2022, 22,194 participants were recruited into the control series of the case–control study on risk factors for SARS-CoV-2 infection. Their complete description is available in Supplementary Table S9. Among them, 2,672 (12.0%) performed a self-test in the 8 days before responding to the questionnaire and 906 (33.9%) of them had a positive result, representing 4.1% of the total control population. However, it is likely that they did not get a positive confirmatory supervised test following their positive self-test, since it would have disqualified them from participating in the control series. Self-test uptake was found to be time-dependent, with a higher uptake during the BA.1-driven epidemic wave in January 2022 than during the BA.2-driven epidemic starting at the beginning of March 2022 (see Supplementary Figure S10).

The most frequent reasons for the control group to self-test were similar to reasons given by cases: history of contact with an infected individual (n = 880, 32.9%) and symptoms (n = 633, 23.7%). Other reasons included: ‘systematic testing’ (n = 398, 14.9%), ‘to see a close relative’ (n = 320, 12.0%) and ‘to work’ (n = 191, 7.1%), see Supplementary Figure S11. The changes in reasons for self-testing throughout the study period are reported in Supplementary Figure S12.

## Discussion

This study of more than 200,000 participants from France showed that the French population quickly and widely adopted self-testing during the first (BA.1) and second (BA.2) SARS-CoV-2 Omicron variant-driven epidemic. The collected data showed that the main reasons for self-testing among SARS-CoV-2-positive persons were symptoms, contact with another infected individual and systematic testing.

The high observed self-test uptake among the controls was consistent with another French survey about testing habits in the general population [36]. The survey showed that 21% of participants had taken a self-test between November 2021 and the end of April 2022 [36]. In the present study, among control participants, self-test uptake in the 8 days prior to questionnaire filling ranged from 8 to 27%, depending on the week of questionnaire filling. Based on this testing rate, one can estimate that during the study period, between 678,000 (0.08 x 67.8/8) and 2.29 million (0.27 x 67.8/8) self-tests were performed each day, without accounting for multiple uses. Between 27 December 2021 and 15 February 2022, self-tests were available for as low as €2 in supermarkets and up to around €5 in pharmacies. French newspapers explained this price gap by differences in market power and supply strategy [37]. Self-tests thus contributed to alleviating the financial burden associated with testing population during the Omicron BA.1 and BA.2 waves. However, contrary to supervised tests, individuals typically had to pay for their self-tests. The introduction of such an out-of-pocket test shifted part of the tax-funded costs associated with population testing to households and may have prevented their wider use due to economic reasons. The decision to only allow pharmacies to sell self-tests in mid-February 2022, which yielded an increase in the average price for self-tests, contributed to a decrease in their accessibility while increasing the financial burden associated with self-testing. We were not aware of any published study trying to estimate the effect of paying out-of-pocket on self-test use. Such a study might, however, be useful to understand if and how this increase in the average price independently contributed to the lower self-test uptake reported during the Omicron BA.2 than during Omicron BA.1 wave among the controls. Alternative explanations are the size of the epidemic wave, smaller for BA.2 compared with BA.1 and possibly epidemic fatigue.

Social, ethnic and geographical inequalities in SARS-CoV-2 testing uptake have already been documented, especially in the United States (US) and the United Kingdom (UK) [38-44]. A study published in 2022 [13] specifically focused on self-test uptake in the US and concluded results consistent with ours, although the study did not go beyond univariable analysis and focused on self-test uptake in the 30 days preceding questionnaire filling. The authors concluded that people aged ≥75 years were less likely to self-test than people aged 18–29 years (3.6% and 5.1%, respectively) and likewise for those with a high school degree or less compared with those with a postgraduate degree (3.5% versus 8.4%, respectively). These studies are part of a broader literature stream studying the interplay between COVID-19 testing behaviour and structural inequalities, such as knowledge barriers, restricted resources and restricted accessibility to test sites [38,44,45]. Our analysis also showed that self-test uptake was significantly linked with professional activities such as being a healthcare worker or having teaching-related activities. For healthcare workers, easy access to free RT-PCR testing at worksite may have prevented them from using self-tests. Meanwhile, the higher likelihood of self-testing among participants with children or those with teaching-related activities in kindergarten, primary or middle school may be explained by the free and organised distribution of self-tests to children who have had contact with another infected child at school (starting mid-January 2022) and to teachers (starting 1 February 2022).

Our study contributes to a better understanding of the possible underlying drivers of self-test uptake among symptomatic SARS-CoV-2 infected individuals who are not aware of any contact with another infected individual. These results may contribute to improving the communication towards individuals with the lowest probability of performing self-tests, hence ultimately driving their behaviour towards a greater uptake. These results may also help find the right balance for self-testing in the context of future pandemics, for individual management when early treatment with antiviral drugs is required for better efficacy [46], or for improving our capacities to mitigate SARS-CoV-2 spread in the future while alleviating the financial burden associated with screening [47,48]. Still, the complete substitution of supervised tests for self-tests would have implications for surveillance and public health response [48,49], epidemic controls due to under-reporting of cases [50] and phylogenetic and phylodynamic approaches, which proved to be useful during the pandemic [51].

The conclusions drawn in this study are, however, limited by a set of features coming from the observational study design. First, data were collected through a self-administered online questionnaire, which may lead to inaccurate answers and occasionally recall bias [52,53]. An additional limitation came from the way we recruited the cases: only SARS-CoV-2 infected individuals detected through supervised tests received an invitation to participate. By design, individuals who did not want to be tested at all, or self-tested but did not confirm their results through supervised tests, were excluded. Such individuals may include those infected through a known contact who did not feel the need for further testing, those with limited access to supervised tests (diagnostic access bias [54]), those who received a false-negative result due to test failure or those who did not want to know their SARS-CoV-2 status to avoid self-isolation. Additional sources of selection bias may come from the Internet-based recruitment, which may decrease the participation of specific groups in the study [55]. As a result, findings may not be generalised to the French adult population who experienced a SARS-CoV-2 infection, although cases were sampled nationwide [56]. This limitation also applies to the selection of controls, whose selection was also influenced by the matching process with cases, resulting in an over-representation of women below 60 years of age compared with the general population (see Supplementary Table S13).

## Conclusions

The COVID-19 pandemic provided a unique opportunity to evaluate the nationwide deployment of self-tests in the context of a public health emergency. While the uptake of self-tests was high, it was not evenly distributed in the population. Thus efforts should be made to increase the use of self-tests among certain groups, such as those over 60 years of age, individuals with lower education level and people not French by birth. Further qualitative studies aiming to understand the barriers associated with self-test uptake in these groups should be conducted.

## Supplementary Data

785000 Supplementary Material

## References

[1] Peeling RW Olliaro PL Boeras DI Fongwen N . Scaling up COVID-19 rapid antigen tests: promises and challenges. Lancet Infect Dis. 2021;21(9):e290-5. 10.1016/S1473-3099(21)00048-7 33636148 PMC7906660

[2] Mercer TR Salit M . Testing at scale during the COVID-19 pandemic. Nat Rev Genet. 2021;22(7):415-26. 10.1038/s41576-021-00360-w 33948037 PMC8094986

[3] Smith RL Gibson LL Martinez PP Ke R Mirza A Conte M Longitudinal assessment of diagnostic test performance over the course of acute SARS-CoV-2 infection. J Infect Dis. 2021;224(6):976-82. 10.1093/infdis/jiab337 34191025 PMC8448437

[4] Larremore DB Wilder B Lester E Shehata S Burke JM Hay JA Test sensitivity is secondary to frequency and turnaround time for COVID-19 screening. Sci Adv. 2021;7(1):eabd5393. 10.1126/sciadv.abd5393 33219112 PMC7775777

[5] Peeling RW, Heymann DL, Teo Y-Y, et al. Diagnostics for COVID-19: Moving from pandemic response to control. The Lancet Published Online First. 2022; (December). 10.1016/S0140-6736(21)02346-1 PMC8687671 34942102

[6] Drain PK . Rapid diagnostic testing for SARS-CoV-2. N Engl J Med. 2022;386(3):264-72. 10.1056/NEJMcp2117115 34995029 PMC8820190

[7] Killingley B Mann AJ Kalinova M Boyers A Goonawardane N Zhou J Safety, tolerability and viral kinetics during SARS-CoV-2 human challenge in young adults. Nat Med. 2022;28(5):1031-41. 10.1038/s41591-022-01780-9 35361992

[8] Baldanti F Ganguly NK Wang G Möckel M O’Neill LA Renz H Choice of SARS-CoV-2 diagnostic test: challenges and key considerations for the future. Crit Rev Clin Lab Sci. 2022;59(7):445-59. 10.1080/10408363.2022.2045250 35289222 PMC8935452

[9] Bubar KM Middleton CE Bjorkman KK Parker R Larremore DB . SARS-CoV-2 transmission and impacts of unvaccinated-only screening in populations of mixed vaccination status. Nat Commun. 2022;13(1):2777. 10.1038/s41467-022-30144-7 35589681 PMC9120147

[10] Mina MJ Parker R Larremore DB . Rethinking covid-19 test sensitivity a strategy for containment. N Engl J Med. 2020;383(22):e120. 10.1056/NEJMp2025631 32997903

[11] Mina MJ Peto TE García-Fiñana M Semple MG Buchan IE . Clarifying the evidence on SARS-CoV-2 antigen rapid tests in public health responses to COVID-19. Lancet. 2021;397(10283):1425-7. 10.1016/S0140-6736(21)00425-6 33609444 PMC8049601

[12] Jean S Burnham CD Chapin K Garner OB Pant Pai N Turabelidze G At-home testing for infectious diseases: The laboratory where you live. Clin Chem. 2021;68(1):19-26. 10.1093/clinchem/hvab198 34969103

[13] Rader B Gertz A Iuliano AD Gilmer M Wronski L Astley CM Use of at-home COVID-19 tests united states, august 23, 2021march 12, 2022. MMWR Morb Mortal Wkly Rep. 2022;71(13):489-94. 10.15585/mmwr.mm7113e1 35358168 PMC8979595

[14] Kepczynski CM Genigeski JA Koski RR Bernknopf AC Konieczny AM Klepser ME . A systematic review comparing at-home diagnostic tests for SARS-CoV-2: Key points for pharmacy practice, including regulatory information. J Am Pharm Assoc (Wash DC). 2021;61(6):666-677.e2. 10.1016/j.japh.2021.06.012 34274214 PMC8196235

[15] Peaper DR, Kerantzas CA, Durant TJS. Advances in molecular infectious diseases testing in the time of COVID-19. Clin Biochem. 2022; (February):S0009-9120(22)00053-4. 10.1016/j.clinbiochem.2022.02.005 PMC8843810 35181291

[16] Haute Autorité de Santé (HAS). Avis numero 2021.0089/AC/SEAP du 30 décembre 2021 du collège de la haute autorité de santé relatif à l’extension de l’utilisation des autotests de détection antigénique du SARS-CoV-2 sur prélèvement nasal chez les personnes-contacts. [Opinion No. 2021.0089/AC/SEAP of 30 December 2021 from the college of the Haute Autorité de santé relating to the extension of the use of self-tests for the antigenic detection of SARS-CoV-2 on nasal swabs in contact persons.] Paris: HAS. [Accessed: 04 Apr 2023]. French. Available from: https://www.has-sante.fr/jcms/p_3307279/fr/avis-n-2021-0089/ac/seap-du-30-decembre-2021-du-college-de-la-haute-autorite-de-sante-relatif-a-l-extension-de-l-utilisation-des-autotests-de-detection-antigenique-du-sars-cov-2-sur-prelevement-nasal-chez-les-personnes-contacts

[17] Galmiche S Charmet T Schaeffer L Paireau J Grant R Chény O Exposures associated with SARS-CoV-2 infection in France: A nationwide online case-control study. Lancet Reg Health Eur. 2021;7:100148. 10.1016/j.lanepe.2021.100148 34124709 PMC8183123

[18] Charmet T Schaeffer L Grant R Galmiche S Chény O Von Platen C Impact of original, B.1.1.7, and B.1.351/P.1 SARS-CoV-2 lineages on vaccine effectiveness of two doses of COVID-19 mRNA vaccines: Results from a nationwide case-control study in France. Lancet Reg Health Eur. 2021;8:100171. 10.1016/j.lanepe.2021.100171 34278372 PMC8277121

[19] Grant R Charmet T Schaeffer L Galmiche S Madec Y Von Platen C Impact of SARS-CoV-2 Delta variant on incubation, transmission settings and vaccine effectiveness: Results from a nationwide case-control study in France. Lancet Reg Health Eur. 2022;13:100278. 10.1016/j.lanepe.2021.100278 34849500 PMC8616730

[20] Gourieroux C Monfort A Trognon A . Pseudo maximum likelihood methods: Applications to poisson models. Econometrica. 1984;52(3):701-20. 10.2307/1913472

[21] Wooldridge JM. M-estimation, nonlinear regression, and quantile regression. In: Econometric analysis of cross section and panel data. Cambridge: The MIT Press; 2010. 397-468. [Accessed: 23 Jan 2023]. Available from: http://www.jstor.org/stable/j.ctt5hhcfr.17

[22] Wooldridge JM. Count, fractional, and other nonnegative responses. In: Econometric analysis of cross section and panel data. Cambridge: The MIT Press; 2010. 723-76. [Accessed: 15 Jan 2023]. Available from: http://www.jstor.org/stable/j.ctt5hhcfr.24

[23] Blackburn ML . The relative performance of poisson and negative binomial regression estimators. Oxf Bull Econ Stat. 2015;77(4):605-16. 10.1111/obes.12074

[24] Leeb H Pötscher BM . Model selection and inference: Facts and fiction. Econom Theory. 2005;21(1). 10.1017/S0266466605050036

[25] Vach W Blettner M . Biased estimation of the odds ratio in case-control studies due to the use of ad hoc methods of correcting for missing values for confounding variables. Am J Epidemiol. 1991;134(8):895-907. 10.1093/oxfordjournals.aje.a116164 1670320

[26] Sterne JAC, White IR, Carlin JB, Spratt M, Royston P, Kenward MG, et al. Multiple imputation for missing data in epidemiological and clinical research: potential and pitfalls. BMJ. 2009;338(jun29 1):b2393-3. 10.1136/bmj.b2393 PMC2714692 19564179

[27] Azur MJ Stuart EA Frangakis C Leaf PJ . Multiple imputation by chained equations: what is it and how does it work? Int J Methods Psychiatr Res. 2011;20(1):40-9. 10.1002/mpr.329 21499542 PMC3074241

[28] Rubin D. Multiple imputation for nonresponse in surveys. Hoboken, N.J: Wiley-Interscience 2004.

[29] R Core Team. R: A language and environment for statistical computing. Vienna, Austria: R Foundation for Statistical Computing 2021. Available from: https://www.R-project.org/

[30] van Buuren S Groothuis-Oudshoorn K . mice: Multivariate imputation by chained equations in r. J Stat Softw. 2011;45(3):1-67. 10.18637/jss.v045.i03

[31] Hughes RA Heron J Sterne JAC Tilling K . Accounting for missing data in statistical analyses: multiple imputation is not always the answer. Int J Epidemiol. 2019;48(4):1294-304. 10.1093/ije/dyz032 30879056 PMC6693809

[32] Lee KJ Tilling KM Cornish RP Little RJA Bell ML Goetghebeur E Framework for the treatment and reporting of missing data in observational studies: The Treatment And Reporting of Missing data in Observational Studies framework. J Clin Epidemiol. 2021;134:79-88. 10.1016/j.jclinepi.2021.01.008 33539930 PMC8168830

[33] Zeileis A Hothorn T . Diagnostic checking in regression relationships. R News. 2002;2:7-10. Available from: https://CRAN.R-project.org/doc/Rnews/

[34] Zeileis A Köll S Graham N . Various versatile variances: An object-oriented implementation of clustered covariances in R. J Stat Softw. 2020;95(1):1-36. 10.18637/jss.v095.i01

[35] Zeileis A . Object-oriented computation of sandwich estimators. J Stat Softw. 2006;16(9):1-16. 10.18637/jss.v016.i09

[36] Santé publique France. Comment évolue l’adhésion des français aux mesures de prévention contre la covid-19? Résultats de la vague 33 de l’enquête CoviPrev. [How is French people’s adherence to preventive measures against Covid-19 changing? Results of wave 33 of the CoviPrev survey]. Paris: Santé publique France. [Accessed: 4 Apr 2023]. French. Available from: https://www.santepubliquefrance.fr/maladies-et-traumatismes/maladies-et-infections-respiratoires/infection-a-coronavirus/documents/enquetes-etudes/comment-evolue-l-adhesion-des-francais-aux-mesures-de-prevention-contre-la-covid-19-resultats-de-la-vague-33-de-l-enquete-coviprev

[37] Serdic L. Autotests: Pourquoi sont-ils beaucoup moins chers en grande surface qu’en pharmacie? [Self-tests: why are they much cheaper in supermarkets than in pharmacies?]. Toulouse: La Dépêche: 2021. [Accessed: 4 Apr 2023]. French. Available from: https://www.ladepeche.fr/2021/12/31/autotests-pourquoi-ils-sont-beaucoup-moins-chers-en-grande-surface-quen-pharmacie-10021356.php

[38] Green MA García-Fiñana M Barr B Burnside G Cheyne CP Hughes D Evaluating social and spatial inequalities of large scale rapid lateral flow SARS-CoV-2 antigen testing in COVID-19 management: An observational study of Liverpool, UK (November 2020 to January 2021). Lancet Reg Health Eur. 2021;6:100107. 10.1016/j.lanepe.2021.100107 34002172 PMC8114854

[39] Holden TM Richardson RAK Arevalo P Duffus WA Runge M Whitney E Geographic and demographic heterogeneity of SARS-CoV-2 diagnostic testing in Illinois, USA, March to December 2020. BMC Public Health. 2021;21(1):1105. 10.1186/s12889-021-11177-x 34107947 PMC8189821

[40] French CE Denford S Brooks-Pollock E Wehling H Hickman M . Low uptake of COVID-19 lateral flow testing among university students: a mixed methods evaluation. Public Health. 2022;204:54-62. 10.1016/j.puhe.2022.01.002 35176622 PMC8755476

[41] Smith LE Potts HW Amlôt R Fear NT Michie S Rubin GJ . Who is engaging with lateral flow testing for COVID-19 in the UK? The COVID-19 Rapid Survey of Adherence to Interventions and Responses (CORSAIR) study. BMJ Open. 2022;12(2):e058060. 10.1136/bmjopen-2021-058060 35144956 PMC8845094

[42] Griffiths D Leder K Collie A . Sociodemographic indicators of COVID-19 testing amongst working-age Australians. Health Promot J Austr. 2021;32(2):361-4. 10.1002/hpja.472 33723869 PMC8250624

[43] Rader B Astley CM Sy KTL Sewalk K Hswen Y Brownstein JS Geographic access to United States SARS-CoV-2 testing sites highlights healthcare disparities and may bias transmission estimates. J Travel Med. 2020;27(7):taaa076. 10.1093/jtm/taaa076 32412064 PMC7239151

[44] Graham MS May A Varsavsky T Sudre CH Murray B Kläser K Knowledge barriers in a national symptomatic-COVID-19 testing programme. PLOS Glob Public Health. 2022;2(1):e0000028. 10.1371/journal.pgph.0000028 36962066 PMC10022193

[45] Vandentorren S Smaïli S Chatignoux E Maurel M Alleaume C Neufcourt L The effect of social deprivation on the dynamic of SARS-CoV-2 infection in France: a population-based analysis. Lancet Public Health. 2022;7(3):e240-9. 10.1016/S2468-2667(22)00007-X 35176246 PMC8843336

[46] Saravolatz LD Depcinski S Sharma M . Molnupiravir and Nirmatrelvir-Ritonavir: Oral Coronavirus Disease 2019 Antiviral Drugs. Clin Infect Dis. 2023;76(1):165-71. 10.1093/cid/ciac180 35245942 PMC9383515

[47] World Health Organisation (WHO). Use of SARS-CoV-2 antigen-detection rapid diagnostic tests for COVID-19 self-testing INTERIM GUIDANCE. Geneva: WHO; 2022.Available from: https://apps.who.int/iris/bitstream/handle/10665/352348/WHO-2019-nCoV-Ag-RDTs-Self-testing-Web-annex-E-2022.1-eng.pdf

[48] European Centre for Disease Prevention and Control (ECDC). ECDC technical report - considerations on the use of self-tests for COVID-19 in the EU/EEA. Stockholm: ECDC; 2021.Available from: https://www.ecdc.europa.eu/sites/default/files/documents/Considerations-for-the-use-of-self-tests-for-COVID-19-in-the-EU-EEA_0.pdf

[49] Beauté J Adlhoch C Bundle N Melidou A Spiteri G . Testing indicators to monitor the COVID-19 pandemic. Lancet Infect Dis. 2021;21(10):1344-5. 10.1016/S1473-3099(21)00461-8 34450053 PMC8384351

[50] Pullano G Di Domenico L Sabbatini CE Valdano E Turbelin C Debin M Underdetection of cases of COVID-19 in France threatens epidemic control. Nature. 2021;590(7844):134-9. 10.1038/s41586-020-03095-6 33348340

[51] Attwood SW Hill SC Aanensen DM Connor TR Pybus OG . Phylogenetic and phylodynamic approaches to understanding and combating the early SARS-CoV-2 pandemic. Nat Rev Genet. 2022;23(9):547-62. 10.1038/s41576-022-00483-8 35459859 PMC9028907

[52] Drews CD Greeland S . The impact of differential recall on the results of case-control studies. Int J Epidemiol. 1990;19(4):1107-12. 10.1093/ije/19.4.1107 2083997

[53] Coughlin SS . Recall bias in epidemiologic studies. J Clin Epidemiol. 1990;43(1):87-91. 10.1016/0895-4356(90)90060-3 2319285

[54] Sackett DL . Bias in analytic research. J Chronic Dis. 1979;32(1-2):51-63. 10.1016/0021-9681(79)90012-2 447779

[55] Bethlehem J . Selection bias in web surveys. Int Stat Rev. 2010;78(2):161-88. 10.1111/j.1751-5823.2010.00112.x

[56] Lu H Cole SR Howe CJ Westreich D . Toward a clearer definition of selection bias when estimating causal effects. Epidemiology. 2022;33(5):699-706. 10.1097/EDE.0000000000001516 35700187 PMC9378569

