# Supplementary material for: SARS-CoV-2 self-test uptake and factors associated with self-testing during Omicron BA.1 and BA.2 waves in France, January to May 2022
Source: Euro Surveill. 2023 May 4;28(18):2200781. doi: 10.2807/1560-7917.ES.2023.28.18.2200781 (PMC10161682; doi:10.2807/1560-7917.ES.2023.28.18.2200781)
Supplement: Supplementary Material [file 22-00781_FONTANET_Supplementary_material.pdf]

Supplementary files

This supplementary material is hosted by *Eurosurveillance* as supporting information alongside the article 'SARS-CoV-2 self-test uptake and factors associated with self-testing during Omicron BA.1 and BA.2 waves in France, January to May 2022', on behalf of the authors, who remain responsible for the accuracy and appropriateness of the content.

The same standards for ethics, copyright, attributions and permissions as for the article apply. Supplements are not edited by *Eurosurveillance* and the journal is not responsible for the maintenance of any links or email addresses provided therein.

Supplementary Figure S1: Weekly number of positive supervised tests, predominantly circulating variant, and policy milestones regarding self-tests, throughout the study period

Following the French National Authority for Health's decisions regarding the use of self-tests for 15+ year olds (2021-03-15) and below 15 (2021-04-26), self-tests were available without medical prescription in pharmacies only, except during a short period in late 2021 - early 2022 where they were also available in supermarkets.

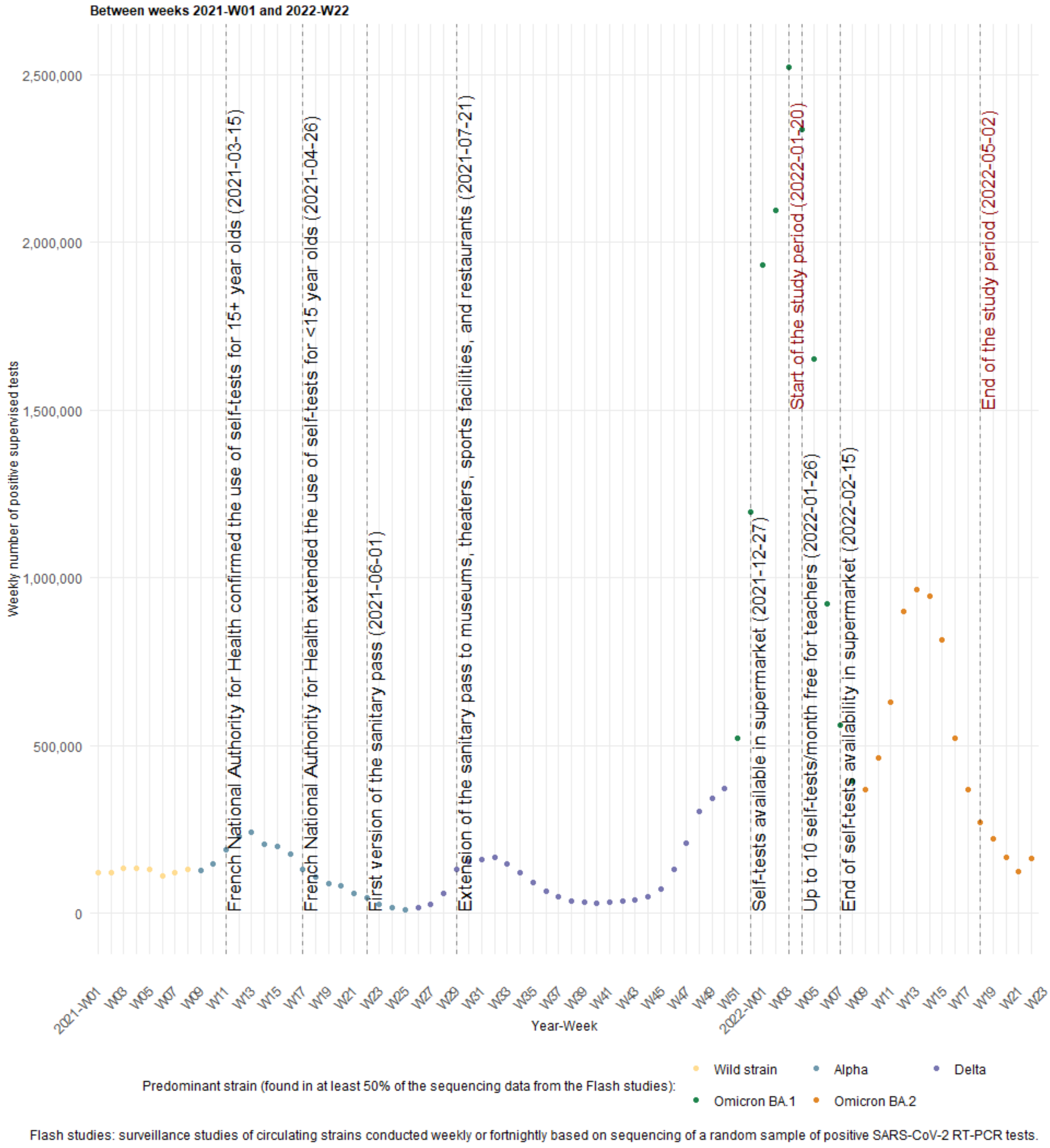

Supplementary Figure S2: Correlation matrix between included explanatory variables

This supplementary figure shows the correlation matrix between all the variables included in the multivariable modeling. All variables were transformed into a set of dummy variable which were further used for producing this matrix. The y-axis considered the variables in the same order as the x axis.

Weeks of symptom onset were removed to keep figure readable.

Variables exhibiting a perfect negative correlation (dark blue square) are binary variables. The diagonal shows the correlation between each variable with itself, hence a perfect positive correlation (red square). The matrix shows that pairwise correlations can be considered weak, ruling out any multicollinearity issue.

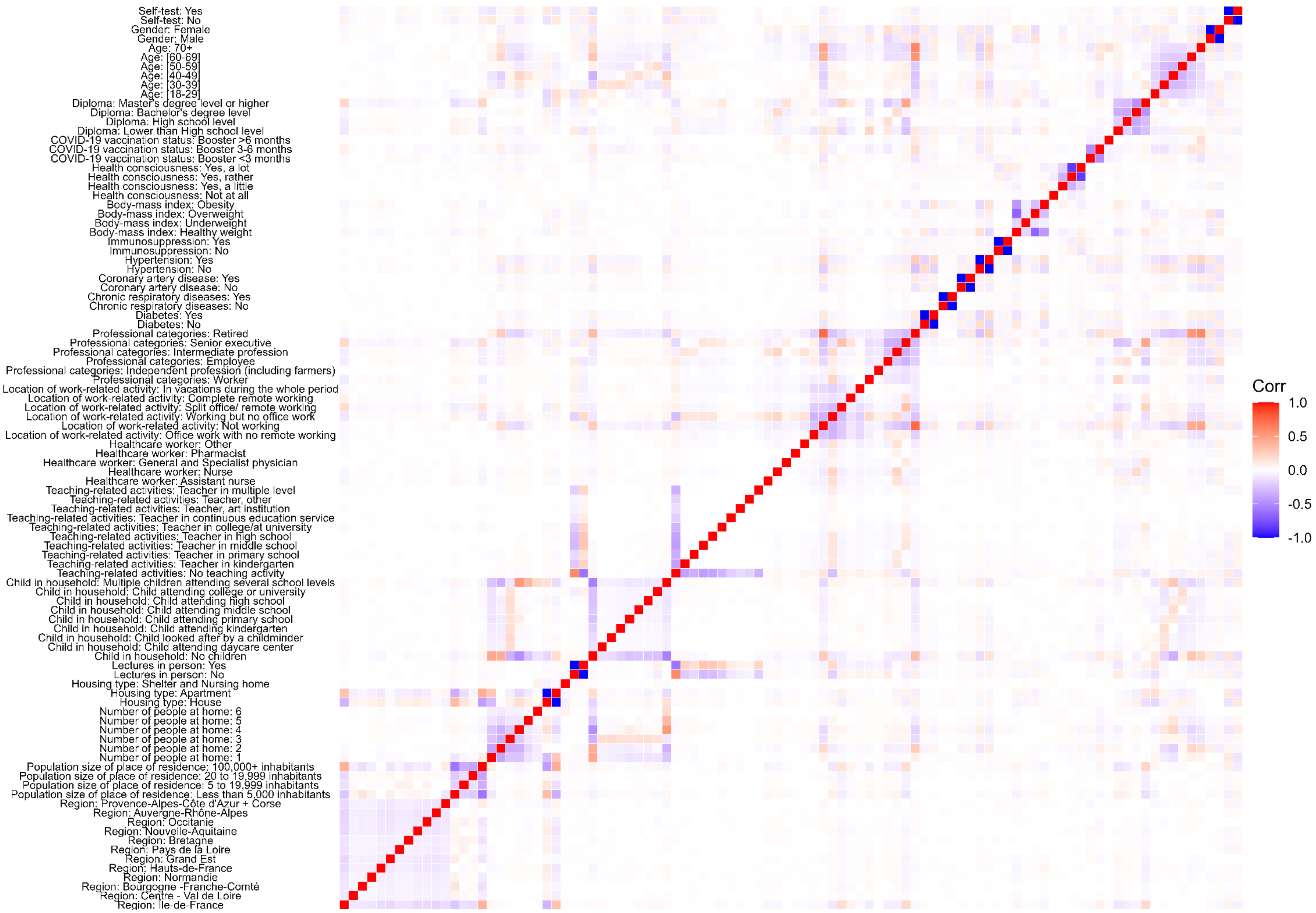
Self-test: Yes
Self-test: No
Gender: Female
Gender: Male
Age: 70+
Age: [60-69]
Age: [50-59]
Age: [40-49]
Age: [30-39]
Age: [18-29]
Diploma: Master's degree level or higher
Diploma: Bachelor's degree level
Diploma: High school level
Diploma: Lower than High school level
COVID-19 vaccination status: Booster >6 months
COVID-19 vaccination status: Booster 3-6 months
COVID-19 vaccination status: Booster <3 months
Health consciousness: Yes, a lot
Health consciousness: Yes, rather
Health consciousness: Yes, a little
Health consciousness: Not at all
Body-mass index: Obesity
Body-mass index: Overweight
Body-mass index: Underweight
Body-mass index: Healthy weight
Immunosuppression: Yes
Immunosuppression: No
Hypertension: Yes
Hypertension: No
Coronary artery disease: Yes
Coronary artery disease: No
Chronic respiratory diseases: Yes
Chronic respiratory diseases: No
Diabetes: Yes
Diabetes: No
Professional categories: Retired
Professional categories: Senior executive
Professional categories: Intermediate profession
Professional categories: Employee
Professional categories: Independent profession (including farmers)
Professional categories: Worker
Location of work-related activity: In vacations during the whole period
Location of work-related activity: Complete remote working
Location of work-related activity: Split office/ remote working
Location of work-related activity: Working but no office work
Location of work-related activity: Not working
Location of work-related activity: Office work with no remote working
Healthcare worker: Other
Healthcare worker: Pharmacist
Healthcare worker: General and Specialist physician
Healthcare worker: Nurse
Healthcare worker: Assistant nurse
Teaching-related activities: Teacher in multiple level
Teaching-related activities: Teacher, other
Teaching-related activities: Teacher, art institution
Teaching-related activities: Teacher in continuous education service
Teaching-related activities: Teacher in college/at university
Teaching-related activities: Teacher in high school
Teaching-related activities: Teacher in middle school
Teaching-related activities: Teacher in primary school
Teaching-related activities: Teacher in kindergarten
Teaching-related activities: No teaching activity
Child in household: Multiple children attending several school levels
Child in household: Child attending college or university
Child in household: Child attending high school
Child in household: Child attending middle school
Child in household: Child attending primary school
Child in household: Child attending kindergarten
Child in household: Child looked after by a childminder
Child in household: Child attending daycare center
Child in household: No children
Lectures in person: Yes
Lectures in person: No
Housing type: Shelter and Nursing home
Housing type: Apartment
Housing type: House
Number of people at home: 6
Number of people at home: 5
Number of people at home: 4
Number of people at home: 3
Number of people at home: 2
Number of people at home: 1
Population size of place of residence: 100,000+ inhabitants
Population size of place of residence: 20 to 19,999 inhabitants
Population size of place of residence: 5 to 19,999 inhabitants
Population size of place of residence: Less than 5,000 inhabitants
Region: Provence-Alpes-Côte d'Azur + Corse
Region: Auvergne-Rhône-Alpes
Region: Occitanie
Region: Nouvelle-Aquitaine
Region: Bretagne
Region: Pays de la Loire
Region: Grand Est
Region: Hauts-de-France
Region: Normandie
Region: Bourgogne -Franche-Comté
Region: Centre - Val de Loire
Region: Ile-de-France
Corr
1.0
0.5
0.0
-0.5
-1.0

Supplementary Table S3: STROBE checklist

The following supplementary table includes the STROBE checklist for cross-sectional studies, as asked by one reviewer.

STROBE Statement—Checklist of items that should be included in reports of ***cross-sectional studies***

	Item No	Recommendation
**Title and abstract**	1	***(a)*** **Indicate the study's design with a commonly used term in the title or the abstract**<br><br>Cross-sectional study, as indicated by the first sentence in the method part from the summary and the STROBE Statement version used for the checklist.<br><br>Also indicated in the first sentence of the last paragraph of the introduction.
		**(*b*) Provide in the abstract an informative and balanced summary of what was done and what was found**<br><br>See the abstract.
**Introduction**		
Background/rationale	2	**Explain the scientific background and rationale for the investigation being reported**<br><br>See penultimate paragraph in the introduction.
Objectives	3	**State specific objectives, including any prespecified hypotheses**<br><br>See last paragraph in the introduction:<br><br>'In this study, we used the cases and the controls series of a case-control study on factors associated with SARS-CoV-2 infection to explore two different aims: 1) The case series, made of individuals who recently had a positive supervised test, was used to estimate the self-test uptake among SARS-CoV-2 infected individuals, and factors associated with self-test uptake among the symptomatic ones; and 2) The control series was used to estimate the self-test uptake among non-infected individuals as a proxy of the self-test background rate in the non-infected population of France.'
**Methods**		
Study design	4	**Present key elements of study design early in the paper**<br><br>See beginning of the last paragraph in the introduction: 'In this study, we used the cases and the controls series of a case-control study on factors associated with SARS-CoV-2 infection to explore two different aims:'<br><br>Also provided in the 'Study setting' subsection.
Setting	5	**Describe the setting, locations, and relevant dates, including periods of recruitment, exposure, follow-up, and data collection**

		See 'Study setting' and 'Population selection' subsections.
Participants	6	**(*a*) Give the eligibility criteria, and the sources and methods of selection of participants**<br><br>See 'Population selection' subsection and Figure 1.
Variables	7	**Clearly define all outcomes, exposures, predictors, potential confounders, and effect modifiers. Give diagnostic criteria, if applicable**<br><br>See last paragraph in 'Study design' and the 'Statistical analysis' subsections.
Data sources/ measurement	8*	**For each variable of interest, give sources of data and details of methods of assessment (measurement). Describe comparability of assessment methods if there is more than one group**<br><br>Provided in the 'Study setting' subsection.
Bias	9	**Describe any efforts to address potential sources of bias**<br><br>See 'Statistical analysis' subsection.
Study size	10	**Explain how the study size was arrived at**<br><br>From the 'statistical analysis' subsection:<br><br>'Since this analysis was not planned at the initiation of the study, we did not calculate a sample size based on an expected increase in self-testing uptake associated with participants' characteristics. The sample size happened to be the number of participants who responded to the questionnaire during the study period and who matched the criteria chosen for the analysis.'
Quantitative variables	11	**Explain how quantitative variables were handled in the analyses. If applicable, describe which groupings were chosen and why**<br><br>Provided in the 'Statistical method' subsection:<br><br>'The only variable that was collected as continuous was age, from which we created six categories based on the cut-offs of previous ComCor analyses. All other variables were qualitative and all of them were also included in past ComCor articles. Pairwise correlations were low, excluding any multicollinearity issue (see Supplementary Figure S2).'
Statistical methods	12	**(*a*) Describe all statistical methods, including those used to control for confounding**<br><br>Confounding was handled by considering within the same model an extensive set of potential confounders (multivariable analysis). See 'Statistical analysis' subsection and Tables 1, 2, 3 for the complete list of variables.
		**(*b*) Describe any methods used to examine subgroups and interactions**

		We did not explore subgroup and interaction.
		**(*c*) Explain how missing data were addressed**<br><br>From the 'Statistical analysis' subsection:<br>'Instead, missing values were handled using multiple imputations by chained equation [..] using all variables included in the model and the outcome.'
		**(*d*) If applicable, describe analytical methods taking account of sampling strategy**<br><br>Not applicable
		**(*e*) Describe any sensitivity analyses**<br><br>From the 'Statistical analysis' subsection:<br>'Sensitivity analysis was performed considering a complete case approach, which is known to be unbiased when the missingness pattern does not depend on the targeted outcome.'
**Results**		
Participants	13*	**(a) Report numbers of individuals at each stage of study—eg numbers potentially eligible, examined for eligibility, confirmed eligible, included in the study, completing follow-up, and analysed**<br><br>Provided in Figure 1
		**(b) Give reasons for non-participation at each stage**<br><br>Provided in Figure 1 and 'Population selection' subsection
		**(c) Consider use of a flow diagram**<br><br>Provided - See Figure 1
Descriptive data	14*	**(a) Give characteristics of study participants (eg demographic, clinical, social) and information on exposures and potential confounders**<br><br>See Tables 1, 2, 3, for analysis of case series and Supplementary Table S8 for the analysis of control series.
		**(b) Indicate number of participants with missing data for each variable of interest**<br><br>See Supplementary Table S4
Outcome data	15*	**Report numbers of outcome events or summary measures**<br><br>See Figure 1, Columns' label in Tables 1, 2, 3 and Supplementary Tables and Figures

Main results	16	**(*a*) Give unadjusted estimates and, if applicable, confounder-adjusted estimates and their precision (eg, 95% confidence interval). Make clear which confounders were adjusted for and why they were included:**<br><br>See Tables 1 to 3.<br>The columns 'Univariable' and 'Multivariable' respectively give the unadjusted and adjusted estimates along with their 95% CI.<br>All variables in those tables were used simultaneously in the same model, as explained in the 'Statistical analysis' subsection and footnote b.
		**(*b*) Report category boundaries when continuous variables were categorized**<br><br>See Table 1, 2, and 3.
		**(*c*) If relevant, consider translating estimates of relative risk into absolute risk for a meaningful time period**<br><br>Not relevant
Other analyses	17	**Report other analyses done—eg analyses of subgroups and interactions, and sensitivity analyses**<br><br>See last part of the 'Results' section for supplementary results on the control series available in the dataset. Corresponding Tables and Figures are reported in supplementary files.
**Discussion**		
Key results	18	**Summarise key results with reference to study objectives**<br><br>See first and second paragraph in the 'Discussion' section, respectively for study aim 2 and 1.
Limitations	19	**Discuss limitations of the study, taking into account sources of potential bias or imprecision. Discuss both direction and magnitude of any potential bias**<br><br>See penultimate paragraph in the 'Discussion' section.
Interpretation	20	**Give a cautious overall interpretation of results considering objectives, limitations, multiplicity of analyses, results from similar studies, and other relevant evidence**<br><br>See first, second and last paragraph from the 'Discussion section'.
Generalisability	21	**Discuss the generalisability (external validity) of the study results**<br><br>See penultimate paragraph in the 'Discussion' section and Supplementary Table S13.
**Other information**		

Funding	22	**Give the source of funding and the role of the funders for the present study and, if applicable, for the original study on which the present article is based**<br><br>See the 'Role of the funding source' section:<br><br>'The funding source had no role in the study design, collection, analysis, and interpretation of data, writing of the report, or decision to submit the paper for publication.'<br><br>See funding information declared during the submission.<br><br>'The study was funded by Institut Pasteur and Research & Action Emerging Infectious Diseases (REACTing). AF's laboratory receives support from the Labex IBEID (ANR-10-LABX-62-IBEID) and the INCEPTION project (PIA/ANR-16-CONV-0005) for studies on emerging viruses. TC is funded by the Fondation de France (Alliance "Tous unis contre le virus'). SG is funded by the INCEPTION project (PIA/ANR-16-CONV-0005).'

*Give information separately for exposed and unexposed groups.

Note: An Explanation and Elaboration article discusses each checklist item and gives methodological background and published examples of transparent reporting. The STROBE checklist is best used in conjunction with this article (freely available on the Web sites of PLoS Medicine at http://www.plosmedicine.org/, Annals of Internal Medicine at http://www.annals.org/, and Epidemiology at http://www.epidem.com/). Information on the STROBE Initiative is available at www.strobe-statement.org.

Supplementary Table S4: Missing values

	Controls		Cases		Symptomatic recruited cases without any history of contact with an infected individual		Self-test uptakers	
**Variable**	**N (22,194)**	**Col %[a]**	**N (179,165)**	**Col %[a]**	**N (75,463)**	**Col %[a]**	**N (44,132)**	**Col %[a]**
French citizenship	88	0.4	950	0.5	438.0	0.6	215.0	0.5
COVID-19 vaccination status	3,734	16.8	13,330	7.4	5,972.0	7.9	3,216.0	7.3
Immunosuppression	137	0.6	1,813	1	767.0	1	414.0	0.9
Comorbidities (except BMI)	247	1.1	2,474	1.4	992.0	1.3	573.0	1.3

[a] Of the total N

Supplementary Table S5: Results for the multivariable analysis for the variable 'week of symptom onset'

This supplementary table displays the results for the variable 'week of symptom onset' in the multivariable analysis.

	Recruited cases[a]		Symptomatic recruited cases who tested for other reasons than contact with another SARS-CoV-2 infected individual[b]		Self-test uptakers[c]		Univariable	Multivariable
**Factor**	**N (179,165)**	**Column %[d]**	**N (75,463)**	**Column %[d]**	**N (44,132)**	**Row %[e]**	**RR [95% CI]**	**RR [95% CI]**
**Week of symptom onset**								
3 January 2022	2,849	1.6	1,017	1.3	543	53.4	Ref	
10 January 2022	12,233	6.8	3,811	5.1	2,102	55.2	1.03 [0.97-1.1]	1.01 [0.95-1.08]
17 January 2022	4,667	2.6	1,427	1.9	834	58.4	1.09 [1.02-1.18]	1.09 [1.02-1.17]
24 January 2022	15,445	8.6	4,897	6.5	2,953	60.3	1.13 [1.06-1.2]	1.13 [1.06-1.2]
31 January 2022	19,442	10.8	6,759	9	4,133	61.1	1.15 [1.08-1.22]	1.14 [1.07-1.21]
7 February 2022	12,646	7.1	4,864	6.4	2,941	60.5	1.13 [1.06-1.2]	1.14 [1.07-1.21]
14 February 2022	7,920	4.4	3,413	4.5	2,080	60.9	1.14 [1.07-1.22]	1.15 [1.08-1.22]
21 February 2022	5,501	3.1	2,522	3.3	1,524	60.4	1.13 [1.06-1.21]	1.14 [1.07-1.22]
28 February 2022	6,628	3.7	3,153	4.2	1,840	58.4	1.09 [1.02-1.17]	1.1 [1.03-1.17]
07 March 2022	10,001	5.6	4,847	6.4	2,904	59.9	1.12 [1.05-1.19]	1.12 [1.06-1.19]
14 March 2022	14,959	8.3	6,937	9.2	4,072	58.7	1.1 [1.03-1.17]	1.1 [1.04-1.17]
21 March 2022	16,949	9.5	7,850	10.4	4,370	55.7	1.04 [0.98-1.11]	1.07 [1-1.13]
28 March 2022	16,742	9.3	7,779	10.3	4,444	57.1	1.07 [1.01-1.14]	1.09 [1.03-1.16]
04 April 2022	16,471	9.2	8,014	10.6	4,709	58.8	1.1 [1.04-1.17]	1.11 [1.05-1.18]
11 April 2022	10,427	5.8	5,060	6.7	2,915	57.6	1.08 [1.01-1.15]	1.1 [1.04-1.17]
18 April 2022	6,159	3.4	3,068	4.1	1,752	57.1	1.07 [1-1.14]	1.1 [1.03-1.18]
25 April 2022	126	0.1	45	0.1	16	35.6	0.67 [0.45-0.99]	0.66 [0.44-0.99]

a: Individuals who tested positive through supervised test (either supervised Ag-RDT or RT-qPCR), who were invited to participate, who agreed to do so, and who were kept in the study population following the population selection process.

b: Multivariable analysis was restricted to participants who did not test because of contact with an infected individual and were symptomatic when they did both their supervised test and their self-test, for self-test uptakers, and their supervised test only for those who did not self-test. Variables with missing values are available in Supplementary Table S5. Multivariable analysis was fitted using all variables displayed in Tables 1 to 3 - socio-demographic, exposure, and health-related characteristics - and week of symptom onset in a single regression model.

c: Among recruited symptomatic cases who did not test because of contact with a SARS-CoV-2 infected individual.

d: Column %: proportion by variable category.

e: Row %: proportion of the second column (b) who performed a self-test according to the variable category.

Supplementary Figure S6: Reasons for self-test uptake among recruited cases by week of symptom onset (symptomatic recruited cases) or supervised test (asymptomatic recruited cases)

The following additional figure displays the reasons for self-test uptake among recruited cases by week of symptom onset (symptomatic recruited cases) or supervised test (asymptomatic recruited cases).

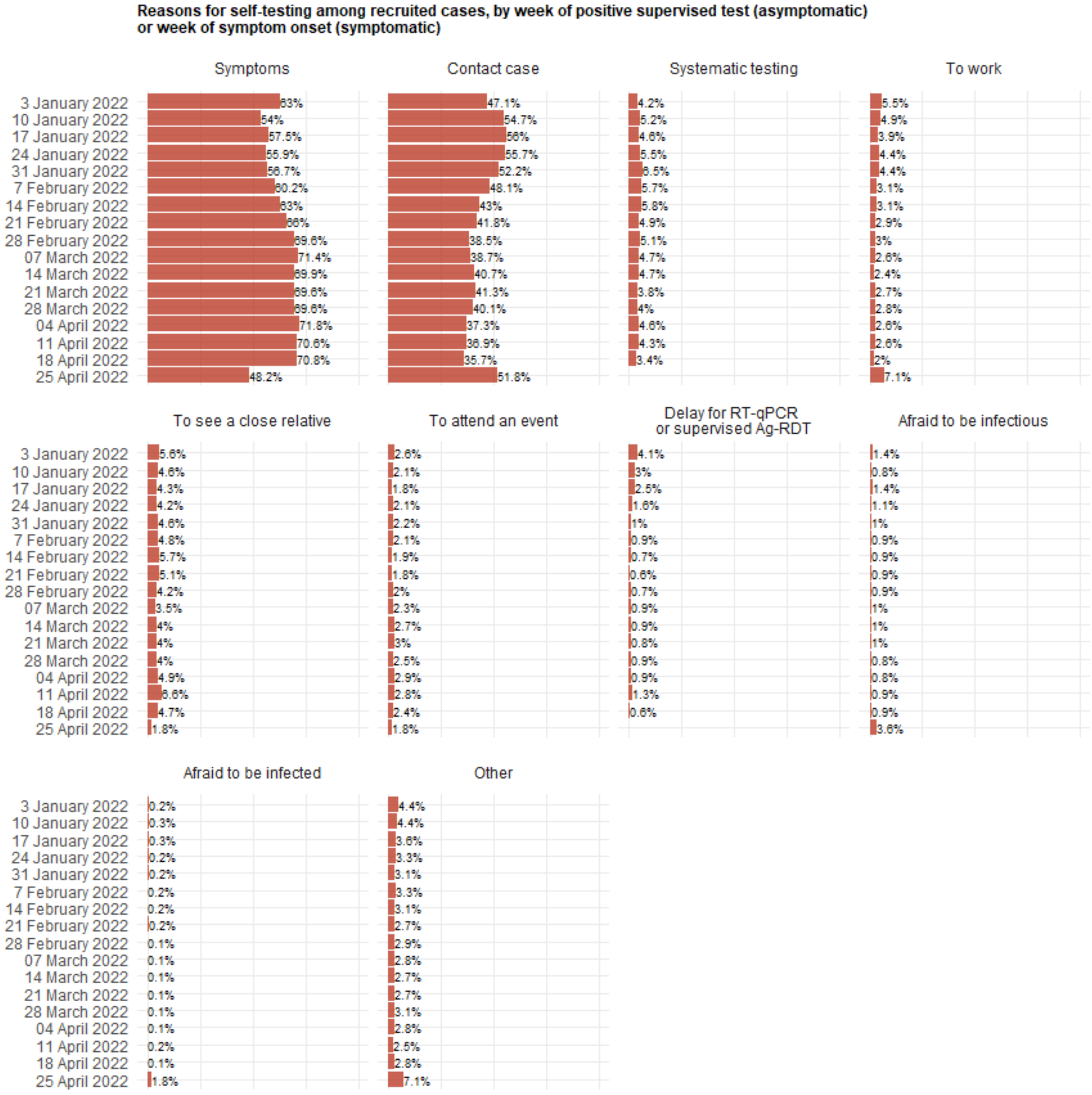

Supplementary Figure S7: Type of supervised test that led to invitation to participate, by comorbidities associated with severe COVID-19

This supplementary figure presents the type of supervised test that led to invitation to participate, by comorbidities associated with severe COVID-19. Note that some participants received confirmation from both an RT-qPCR and a supervised Ag-RDT.

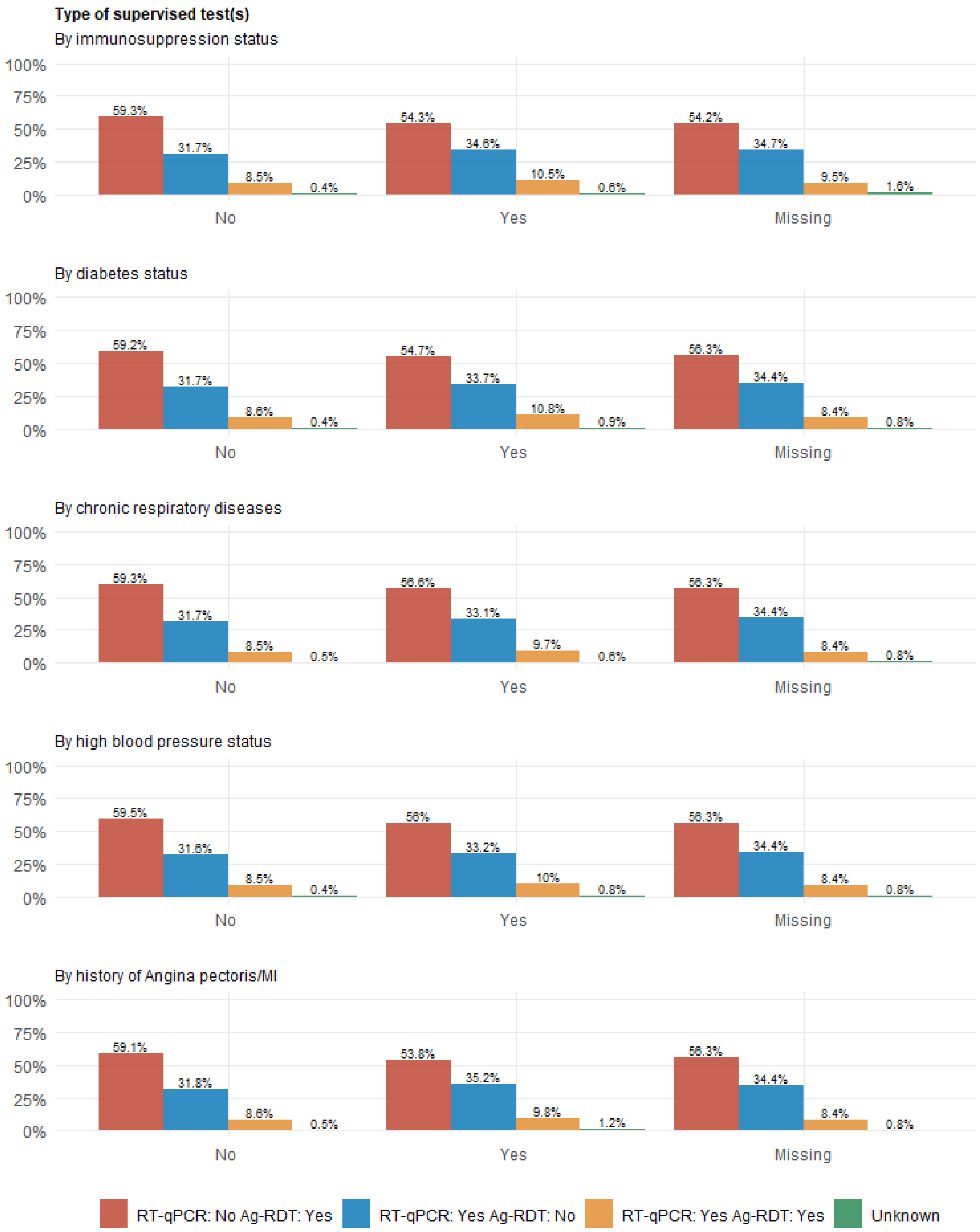

Supplementary Table S8: Comparison between multiple imputations and complete case for the multivariable analysis

This supplementary table compares the results obtained from the multiple imputation approach with these when considering only individuals without any missing values (complete case).

	Symptomatic recruited cases who tested for other reasons than contact with another SARS-CoV-2 infected individual[a]		Self-test uptakers[b]		Multiple imputation[c]		Complete case[d]	
**Factor**	**N (75,463)**	**Column %[e]**	**N (44,132)**	**Row %[f]**	**RR [95% CI]**	**P-value**	**RR [95% CI]**	**P-value**
**Female**	53,293	70.6	31,665	59.4	1.03 [1.01-1.04]	<0.01	1.02 [1.01-1.04]	<0.01
**Age**								
[18-29]	7,479	9.9	4,061	54.3			Ref	
[30-39]	15,434	20.5	9,598	62.2	1.02 [1-1.05]	0.07	1.02 [1-1.05]	0.1
[40-49]	18,669	24.7	12,051	64.6	1.02 [1-1.05]	0.07	1.02 [1-1.05]	0.09
[50-59]	17,992	23.8	10,503	58.4	1 [0.97-1.03]	0.97	1 [0.97-1.02]	0.74
[60-69]	10,624	14.1	5,517	51.9	0.93 [0.9-0.97]	<0.01	0.94 [0.9-0.97]	<0.01
70+	5,265	7	2,402	45.6	0.83 [0.79-0.88]	<0.01	0.84 [0.79-0.88]	<0.01
**Diploma**								
Lower than High school level	13,401	17.8	6,915	51.6			Ref	
High school level	14,201	18.8	8,079	56.9	1.07 [1.05-1.09]	<0.01	1.07 [1.04-1.09]	<0.01
Bachelor's degree level	28,201	37.4	17,335	61.5	1.12 [1.1-1.15]	<0.01	1.12 [1.1-1.15]	<0.01
Master's degree level or higher	19,660	26.1	11,803	60	1.14 [1.11-1.16]	<0.01	1.14 [1.11-1.17]	<0.01
**French citizenship**								
Yes, by birth	69,849	92.6	41,311	59.1			Ref	
No	2,426	3.2	1,219	50.2	0.9 [0.87-0.94]	<0.01	0.9 [0.86-0.94]	<0.01
Yes, by naturalisation, wedding, etc	2,750	3.6	1,387	50.4	0.89 [0.86-0.93]	<0.01	0.9 [0.87-0.94]	<0.01
**COVID-19 vaccination status**								
Unvaccinated, no history of past infection	4,433	5.9	2,641	59.6			Ref	
Unvaccinated, history of past infection	471	0.6	284	60.3	1.03 [0.96-1.11]	0.43	1.01 [0.93-1.09]	0.88
Incomplete primary vaccination series	211	0.3	116	55	0.95 [0.85-1.06]	0.37	0.95 [0.84-1.07]	0.39
Primary vaccination series <3 months	4,814	6.4	2,867	59.6	0.96 [0.93-1]	0.03	0.96 [0.93-0.99]	0.01
Primary vaccination series 3-6 months	9,626	12.8	5,409	56.2	0.94 [0.91-0.97]	<0.01	0.94 [0.91-0.97]	<0.01
Primary vaccination series >6 months	3,842	5.1	2,168	56.4	0.95 [0.92-0.98]	<0.01	0.94 [0.91-0.98]	<0.01
Booster <3 months	24,093	31.9	14,757	61.2	0.98 [0.96-1.01]	0.18	0.98 [0.95-1]	0.1
Booster 3-6 months	21,475	28.5	12,397	57.7	0.99 [0.96-1.02]	0.4	0.98 [0.96-1.01]	0.29
Booster >6 months	526	0.7	277	52.7	1.05 [0.97-1.14]	0.26	1.05 [0.96-1.14]	0.32
**Health consciousness**								
Not at all	338	0.4	179	53			Ref	
Yes, a little	5,381	7.1	3,225	59.9	1.09 [0.99-1.21]	0.09	1.15 [1.02-1.28]	0.02
Yes, rather	44,674	59.2	26,646	59.6	1.09 [0.99-1.21]	0.08	1.14 [1.02-1.28]	0.02
Yes, a lot	25,070	33.2	14,082	56.2	1.06 [0.96-1.17]	0.26	1.11 [0.99-1.24]	0.07
**Body-mass index**								
<18.5	38,415	50.9	22,425	58.4			Ref	
[18.5-25[	2,202	2.9	1,240	56.3	0.98 [0.94-1.01]	0.2	0.97 [0.94-1.01]	0.19
[25-30]	22,586	29.9	13,225	58.5	1.02 [1-1.03]	0.01	1.02 [1-1.03]	0.01
>30	12,260	16.2	7,242	59.1	1.02 [1.01-1.04]	0.01	1.03 [1.01-1.05]	<0.01
**Immunosuppression**	3,829	5.1	1,990	52	0.94 [0.91-0.97]	<0.01	0.94 [0.91-0.97]	<0.01
**Hypertension**	8,989	11.9	4,930	54.8	1.02 [1-1.04]	0.05	1.01 [0.99-1.04]	0.24
**Coronary artery disease**	884	1.2	482	54.5	1.07 [1-1.13]	0.04	1.06 [1-1.13]	0.06
**Chronic respiratory diseases**	6,336	8.4	3,614	57	1 [0.97-1.02]	0.73	1 [0.98-1.02]	0.87
**Diabetes**	2,513	3.3	1,303	51.9	0.97 [0.93-1.01]	0.1	0.97 [0.93-1.02]	0.23
**Professional categories**								
Worker	3,207	4.2	1,753	54.7			Ref	
Independent profession (including farmers)	2,205	2.9	1,313	59.5	1.07 [1.02-1.12]	0.01	1.07 [1.02-1.12]	0.01
Employee	14,482	19.2	8,527	58.9	1.05 [1.01-1.09]	0.01	1.04 [1-1.08]	0.03
Intermediate profession	16,081	21.3	9,939	61.8	1.07 [1.03-1.11]	<0.01	1.07 [1.03-1.11]	<0.01
Senior executive	22,704	30.1	14,124	62.2	1.08 [1.04-1.12]	<0.01	1.07 [1.03-1.12]	<0.01
Unemployed or inactive people	4,508	6	2,402	53.3	1.01 [0.97-1.06]	0.53	1.01 [0.96-1.06]	0.67
Retired	12,276	16.3	6,074	49.5	1.08 [1.03-1.14]	<0.01	1.07 [1.01-1.13]	0.02
**Healthcare worker**								
Not healthcare professional	66,918	88.7	39,421	58.9			Ref	

Factor	Symptomatic recruited cases who tested for other reasons than contact with another SARS-CoV-2 infected individual[a]		Self-test uptakers[b]		Multiple imputation[c]		Complete case[d]	
	N (75,463)	Column %[e]	N (44,132)	Row %[f]	RR [95% CI]	P-value	RR [95% CI]	P-value
Administrative/Management staff	1,197	1.6	642	53.6	0.86 [0.82-0.91]	<0.01	0.86 [0.81-0.91]	<0.01
Assistant nurse	958	1.3	520	54.3	0.87 [0.82-0.93]	<0.01	0.88 [0.83-0.94]	<0.01
Nurse	2,000	2.7	1,153	57.6	0.87 [0.83-0.9]	<0.01	0.87 [0.83-0.91]	<0.01
General and Specialist physician	630	0.8	310	49.2	0.77 [0.71-0.84]	<0.01	0.77 [0.71-0.84]	<0.01
Pharmacist	534	0.7	292	54.7	0.85 [0.79-0.92]	<0.01	0.84 [0.77-0.91]	<0.01
Other	3,226	4.3	1,794	55.6	0.89 [0.86-0.92]	<0.01	0.88 [0.86-0.91]	<0.01
**Location of work-related activity**								
Office work with no remote working	15,074	20	9,163	60.8			Ref	
Not working	16,240	21.5	8,230	50.7	0.96 [0.93-0.99]	<0.01	0.97 [0.94-1]	0.04
Working but no office work	22,051	29.2	13,497	61.2	1 [0.99-1.02]	0.67	1.01 [0.99-1.03]	0.49
Split office/ remote working	12,189	16.2	7,404	60.7	1 [0.98-1.02]	0.83	1 [0.98-1.02]	0.74
Complete remote working	2,930	3.9	1,709	58.3	0.97 [0.94-1]	0.07	0.97 [0.94-1.01]	0.11
In vacations during the whole period	6,979	9.2	4,129	59.2	0.98 [0.96-1.01]	0.19	0.99 [0.96-1.01]	0.24
**Teaching-related activities**								
No teaching activity	71,136	94.3	40,725	57.2			Ref	
Teacher in kindergarten	764	1	658	86.1	1.28 [1.24-1.33]	<0.01	1.28 [1.23-1.33]	<0.01
Teacher in primary school	553	0.7	464	83.9	1.24 [1.19-1.3]	<0.01	1.25 [1.19-1.31]	<0.01
Teacher in middle school	808	1.1	675	83.5	1.25 [1.2-1.3]	<0.01	1.26 [1.21-1.31]	<0.01
Teacher in high school	722	1	584	80.9	1.22 [1.17-1.28]	<0.01	1.23 [1.18-1.29]	<0.01
Teacher in college/at university	462	0.6	305	66	1.08 [1.01-1.16]	0.03	1.07 [1-1.16]	0.05
Teacher in continuous education service	214	0.3	131	61.2	0.97 [0.87-1.08]	0.58	0.97 [0.87-1.08]	0.6
Teacher, art institution	103	0.1	65	63.1	1.08 [0.94-1.25]	0.28	1.07 [0.92-1.25]	0.37
Teacher, other	178	0.2	111	62.4	1.01 [0.9-1.12]	0.9	1.04 [0.93-1.16]	0.52
Teacher in multiple level	523	0.7	414	79.2	1.23 [1.17-1.29]	<0.01	1.23 [1.17-1.29]	<0.01
**Child in household**								
No children	43,808	58.1	23,145	52.8			Ref	
Child attending daycare center	1,024	1.4	592	57.8	1 [0.94-1.06]	0.95	0.99 [0.94-1.05]	0.8
Child looked after by a childminder	1,153	1.5	742	64.3	1.07 [1.02-1.12]	0.01	1.06 [1.01-1.11]	0.02
Child attending kindergarten	2,123	2.8	1,404	66.1	1.13 [1.09-1.17]	<0.01	1.13 [1.09-1.17]	<0.01
Child attending primary school	3,597	4.8	2,505	69.6	1.17 [1.14-1.2]	<0.01	1.17 [1.13-1.2]	<0.01
Child attending middle school	3,128	4.1	2,020	64.6	1.09 [1.05-1.12]	<0.01	1.09 [1.05-1.12]	<0.01
Child attending high school	3,549	4.7	2,252	63.5	1.07 [1.03-1.1]	<0.01	1.06 [1.02-1.09]	<0.01
Child attending college or university	3,475	4.6	2,046	58.9	1.02 [0.98-1.05]	0.32	1.01 [0.98-1.05]	0.48
Multiple children attending several school levels	13,606	18	9,426	69.3	1.14 [1.11-1.17]	<0.01	1.13 [1.1-1.17]	<0.01
**Lectures in person**	6,138	8.1	4,250	69.2	Lectures in person	<0.01	Lectures in person	<0.01
**Housing type**								
House	46,827	62.1	29,506	63			Ref	
Apartment	28,441	37.7	14,554	51.2	0.89 [0.88-0.91]	<0.01	0.9 [0.88-0.91]	<0.01
Shelter and Nursing home	195	0.3	72	36.9	0.7 [0.59-0.84]	<0.01	0.72 [0.59-0.88]	<0.01
**Number of people at home**								
1	16,491	21.9	8,004	48.5			Ref	
2	26,714	35.4	14,981	56.1	1.11 [1.09-1.13]	<0.01	1.11 [1.09-1.13]	<0.01
3	14,115	18.7	8,818	62.5	1.11 [1.08-1.14]	<0.01	1.11 [1.08-1.14]	<0.01
4	13,276	17.6	9,155	69	1.14 [1.1-1.17]	<0.01	1.14 [1.1-1.17]	<0.01
5	3,724	4.9	2,481	66.6	1.1 [1.06-1.14]	<0.01	1.1 [1.06-1.15]	<0.01
6+	1,143	1.5	693	60.6	1.07 [1.01-1.13]	0.02	1.08 [1.02-1.14]	0.01
**Population size of place of residence**								
Less than 5,000 inhabitants	19,316	25.6	12,254	63.4			Ref	
5 to 19,999 inhabitants	8,082	10.7	5,071	62.7	1 [0.98-1.02]	0.71	1 [0.98-1.02]	0.69
20 to 19,999 inhabitants	9,625	12.8	5,827	60.5	1 [0.98-1.02]	0.73	1 [0.98-1.02]	0.82
100,000+ inhabitants	38,440	50.9	20,980	54.6	0.95 [0.93-0.96]	<0.01	0.94 [0.93-0.96]	<0.01
**Region**								
Ile-de-France	15,003	19.9	7,937	52.9			Ref	
Centre - Val de Loire	2,724	3.6	1,771	65	1.12 [1.09-1.16]	<0.01	1.12 [1.08-1.16]	<0.01
Bourgogne -Franche-Comté	3,228	4.3	1,931	59.8	1.06 [1.02-1.09]	<0.01	1.06 [1.02-1.1]	<0.01
Normandie	3,478	4.6	2,162	62.2	1.08 [1.04-1.11]	<0.01	1.08 [1.05-1.12]	<0.01
Hauts-de-France	6,713	8.9	3,676	54.8	0.95 [0.93-0.98]	<0.01	0.96 [0.93-0.99]	0.01
Grand Est	7,513	10	4,398	58.5	1.04 [1.01-1.06]	<0.01	1.04 [1.01-1.06]	0.01
Pays de la Loire	3,991	5.3	2,645	66.3	1.15 [1.12-1.18]	<0.01	1.15 [1.11-1.18]	<0.01

	Symptomatic recruited cases who tested for other reasons than contact with another SARS-CoV-2 infected individual[a]		Self-test uptakers[b]		Multiple imputation[c]		Complete case[d]	
**Factor**	**N (75,463)**	**Column %[e]**	**N (44,132)**	**Row %[f]**	**RR [95% CI]**	**P-value**	**RR [95% CI]**	**P-value**
Bretagne	3,931	5.2	2,531	64.4	1.11 [1.08-1.14]	<0.01	1.11 [1.07-1.14]	<0.01
Nouvelle-Aquitaine	6,706	8.9	4,220	62.9	1.11 [1.08-1.14]	<0.01	1.11 [1.08-1.14]	<0.01
Occitanie	7,279	9.6	4,300	59.1	1.05 [1.02-1.07]	<0.01	1.05 [1.02-1.08]	<0.01
Auvergne-Rhône-Alpes	9,028	12	5,506	61	1.1 [1.07-1.12]	<0.01	1.1 [1.07-1.12]	<0.01
PACA and Corse	5,869	7.8	3,055	52	0.97 [0.95-1]	0.07	0.97 [0.94-1]	0.05
**Week of symptom onset**								
3 January 2022	1,017	1.3	543	53.4		Ref		
10 January 2022	3,811	5.1	2,102	55.2	1.01 [0.95-1.08]	0.68	1.03 [0.97-1.11]	0.32
17 January 2022	1,427	1.9	834	58.4	1.09 [1.02-1.17]	0.02	1.1 [1.02-1.19]	0.01
24 January 2022	4,897	6.5	2,953	60.3	1.13 [1.06-1.2]	<0.01	1.14 [1.07-1.22]	<0.01
31 January 2022	6,759	9	4,133	61.1	1.14 [1.07-1.21]	<0.01	1.16 [1.09-1.24]	<0.01
7 February 2022	4,864	6.4	2,941	60.5	1.14 [1.07-1.21]	<0.01	1.16 [1.08-1.23]	<0.01
14 February 2022	3,413	4.5	2,080	60.9	1.15 [1.08-1.22]	<0.01	1.17 [1.09-1.25]	<0.01
21 February 2022	2,522	3.3	1,524	60.4	1.14 [1.07-1.22]	<0.01	1.16 [1.08-1.24]	<0.01
28 February 2022	3,153	4.2	1,840	58.4	1.1 [1.03-1.17]	<0.01	1.13 [1.05-1.21]	<0.01
07 March 2022	4,847	6.4	2,904	59.9	1.12 [1.06-1.19]	<0.01	1.14 [1.06-1.21]	<0.01
14 March 2022	6,937	9.2	4,072	58.7	1.1 [1.04-1.17]	<0.01	1.12 [1.06-1.2]	<0.01
21 March 2022	7,850	10.4	4,370	55.7	1.07 [1-1.13]	0.03	1.1 [1.03-1.17]	<0.01
28 March 2022	7,779	10.3	4,444	57.1	1.09 [1.03-1.16]	<0.01	1.12 [1.05-1.19]	<0.01
04 April 2022	8,014	10.6	4,709	58.8	1.11 [1.05-1.18]	<0.01	1.14 [1.07-1.21]	<0.01
11 April 2022	5,060	6.7	2,915	57.6	1.1 [1.04-1.17]	<0.01	1.13 [1.06-1.21]	<0.01
18 April 2022	3,068	4.1	1,752	57.1	1.1 [1.03-1.18]	<0.01	1.12 [1.05-1.2]	<0.01
25 April 2022	45	0.1	16	35.6	0.66 [0.44-0.99]	0.04	0.71 [0.46-1.07]	0.1

a: Multivariable analysis was restricted to participants who did not test because of contact with an infected individual and were symptomatic when they did both their supervised test and their self-test, for self-test uptakers, and their supervised test only for those who did not self-test. Variables with missing values are available in Supplementary Table S4. Multivariable analysis was fitted using all the variables from the table in a single regression model.

b: Among the sample used for the multivariable analysis.

c: Estimates with rows with missing values imputed.

d: Estimates with rows with missing values deleted.

e: Column %: proportion by variable category.

f: Row %: proportion of the column (a) who performed a self-test according to the variable category.

Supplementary Table S9: Controls' characteristics

These supplementary tables provide additional information on controls' characteristics.

**Sociodemographic variables**		
**Variables**	**N (22,194)**	**% col**
**Gender**		
Male	9,159	41.3
Female	13,035	58.7
**Age**		
[18-29]	1,855	8.4
[30-39]	3,938	17.7
[40-49]	5,297	23.9
[50-59]	4,734	21.3
[60-69]	4,051	18.2
70+	2,319	10.4
**Diploma**		
Lower than High school level	5,126	23.1
High school level	5,660	25.5
Bachelor's degree level	8,245	37.1
Master's degree level or higher	3,163	14.2
**French citizenship**		
Yes, by birth	21,173	95.4
No	339	1.5
Yes, by naturalisation, wedding, etc	594	2.7
Don't want to answer/Unknown	88	0.4
**Professional categories**		
Worker	1,317	5.9
Independent profession (including farmers)	892	4
Employee	4,877	22
Intermediate profession	3,871	17.4
Senior executive	3,634	16.4
Unemployed or inactive people	2,069	9.3
Retired	5,534	24.9
**Population size of place of residence**		
Less than 5,000 inhabitants	6,233	28.1
5 to 19,999 inhabitants	2,615	11.8
20 to 19,999 inhabitants	3,043	13.7
100,000+ inhabitants	10,303	46.4
**Region**		
Île-de-France	3,679	16.6
Centre - Val de Loire	851	3.8
Bourgogne -Franche-Comté	890	4
Normandie	992	4.5
Hauts-de-France	2,044	9.2
Grand Est	2,013	9.1
Pays de la Loire	1,378	6.2
Bretagne	1,580	7.1
Nouvelle-Aquitaine	2,168	9.8
Occitanie	2,304	10.4
Auvergne-Rhône-Alpes	2,583	11.6
PACA and Corse	1,712	7.7

Exposure-related variables		
**Variables**	**N (22,194)**	**% col**
**Healthcare worker**		
Not healthcare professional	20,751	93.5
Administrative/Management staff	252	1.1
Assistant nurse	208	0.9
Nurse	315	1.4
General and Specialist physician	37	0.2
Pharmacist	75	0.3
Other	556	2.5
**Location of work-related activity**		
Office work with no remote working	3,630	16.4
Not working	7,965	35.9
Working but no office work	5,529	24.9
Split office/ remote working	2,692	12.1
Complete remote working	717	3.2
In vacations during the whole period	1,660	7.5
**Teaching-related activities**		
No teaching activity	21,245	95.7
Teacher in kindergarten	159	0.7
Teacher in primary school	70	0.3
Teacher in middle school	177	0.8
Teacher in high school	166	0.8
Teacher in college/at university	90	0.4
Teacher in continuous education service	62	0.3
Teacher, art institution	16	0.1
Teacher, other	88	0.4
Teacher in multiple level	121	0.6
**Child in household**		
No children	13,608	61.3
Child attending daycare center	179	0.8
Child looked after by a childminder	219	1
Child attending kindergarten	593	2.7
Child attending primary school	1,046	4.7
Child attending middle school	937	4.2
Child attending high school	1,032	4.7
Child attending college or university	1,039	4.7
Multiple children attending several school levels	3,541	15.9
**Lectures in person**		
No	20,717	93.3
**Lectures in person**		
**Housing type**		
House	14,340	64.6
Apartment	7,786	35.1
Shelter and Nursing home	68	0.3
**Number of people at home**		
1	4,556	20.5
2	8,438	38
3	3,936	17.7
4	3,676	16.6
5	1,226	5.5
6+	362	1.6

Health-related variables		
**Variables**	**N (22,194)**	**% col**
**COVID-19 vaccination status**		
Unvaccinated, no history of past infection	1,621	7.3
Unvaccinated, history of past infection	388	1.8
Incomplete primary vaccination series	45	0.2
Primary vaccination series <3 months	1,629	7.3
Primary vaccination series 3-6 months	2,607	11.8
Primary vaccination series >6 months	1,619	7.3
Booster <3 months	5,646	25.4
Booster 3-6 months	4,771	21.5
Booster >6 months	134	0.6
Don't want to answer/Unknown	3,734	16.8
**Health consciousness**		
Not at all	174	0.8
Yes, a little	2,439	11
Yes, rather	13,672	61.6
Yes, a lot	5,909	26.6
**Body-mass index**		
<18.5	9,918	44.7
[18.5-25[	827	3.7
[25-30]	7,178	32.3
>30	4,271	19.2
**Immunosuppression**		
No	20,965	94.5
**Immunosuppression**		
Don't want to answer/Unknown	137	0.6
**Hypertension**		
No	18,669	84.1
**Hypertension**		
**Coronary artery disease**		
No	21,905	98.7
**Coronary artery disease**		
**Chronic respiratory diseases**		
No	20,559	92.6
**Chronic respiratory diseases**		
**Diabetes**		
No	20,797	93.7
**Diabetes**		

Other		
**Variables**	**N (22,194)**	**% col**
**Week of questionnaire completion**		
3 January 2022	0	0
10 January 2022	0	0
17 January 2022	1,282	5.8
24 January 2022	49	0.2
31 January 2022	598	2.7
7 February 2022	410	1.9
14 February 2022	360	1.6
21 February 2022	585	2.6
28 February 2022	946	4.3
07 March 2022	774	3.5
14 March 2022	9,426	42.5
21 March 2022	897	4
28 March 2022	1,411	6.4
04 April 2022	2,505	11.3
11 April 2022	698	3.1
18 April 2022	1,812	8.2
25 April 2022	441	2

Supplementary Figure S10: Share of controls that self-tested during the 8 days preceding the questionnaire completion, by week of questionnaire completion

This supplementary figure provides the share of controls that self-tested during the 8 days preceding the questionnaire completion, by week of questionnaire completion

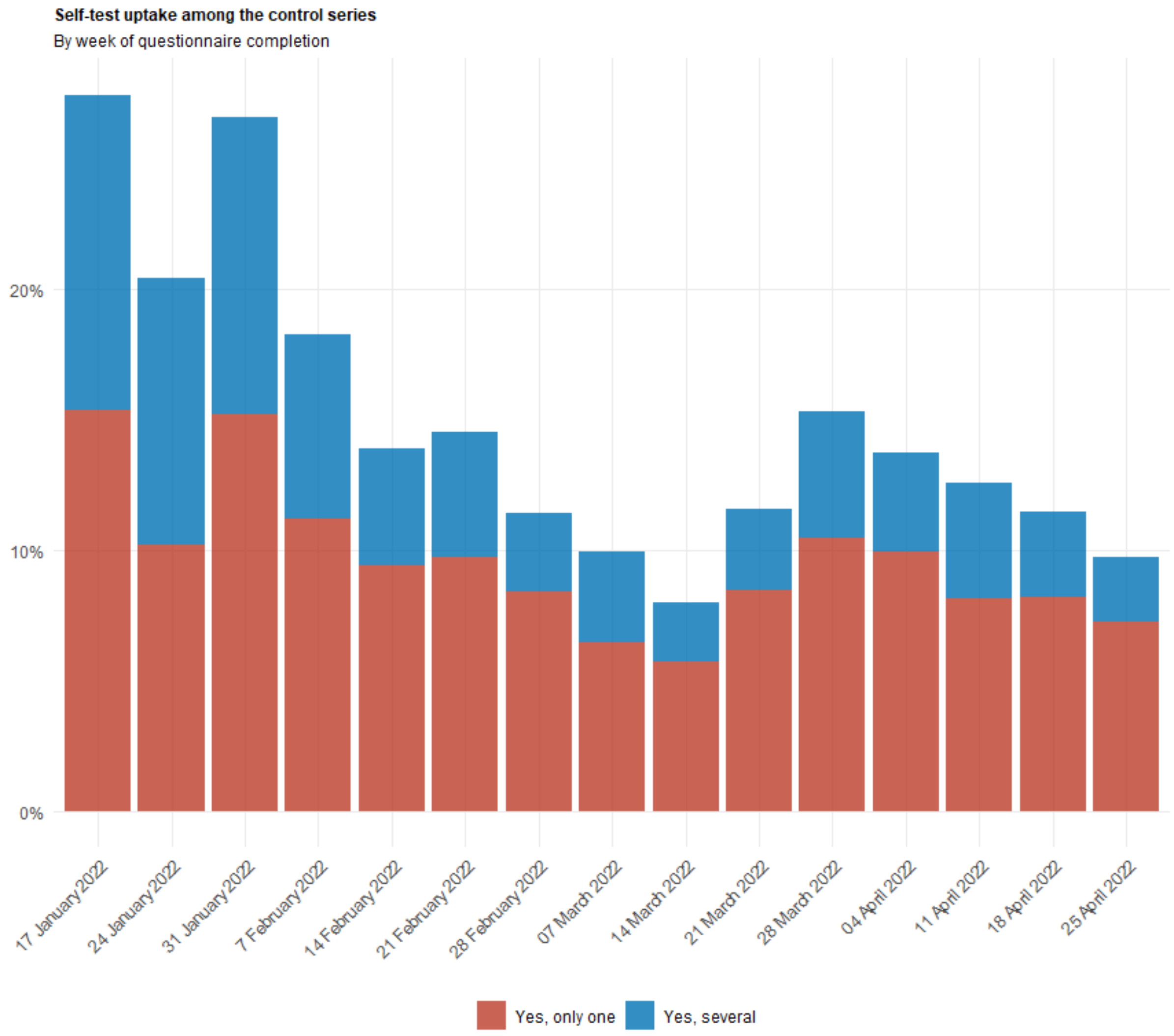

Supplementary Figure S11: Reasons for self-test uptake in the control series

This supplementary figure shows the reasons for self-test uptake in the control series.

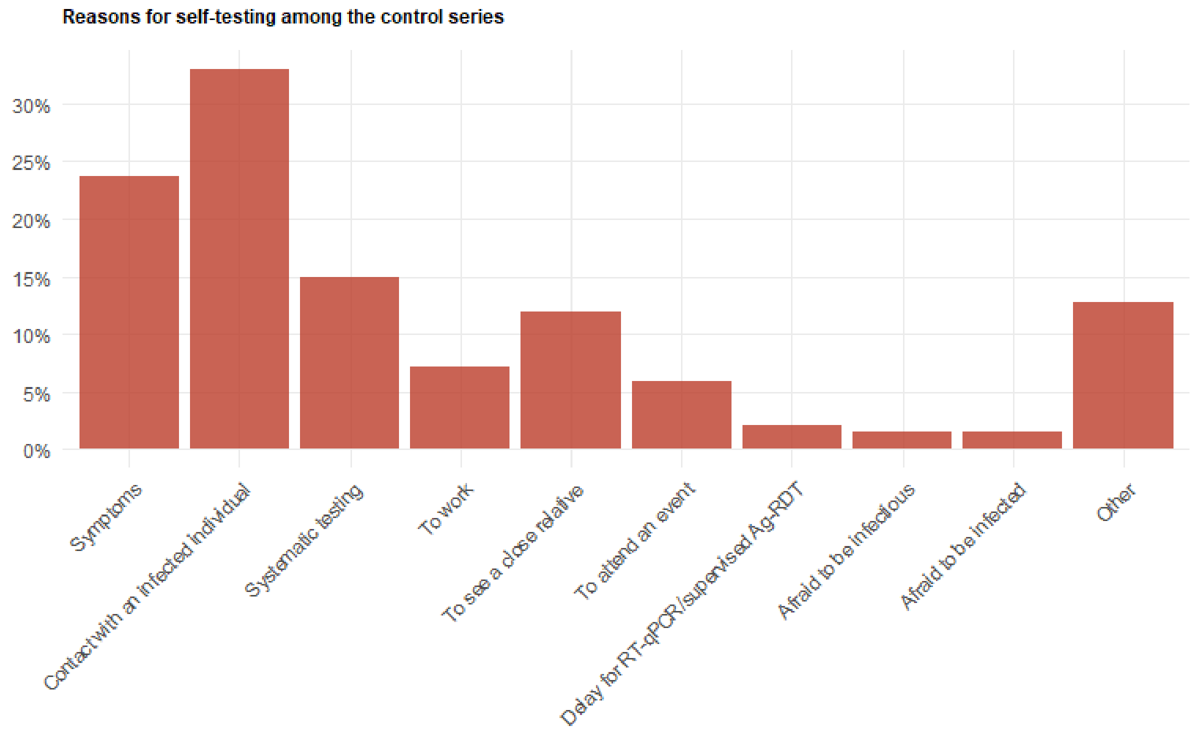

Supplementary Figure S12: Reasons for self-test uptake in the control series, by week of questionnaire completion

This supplementary figure shows the reasons for self-test uptake in the control series, by week of questionnaire completion.

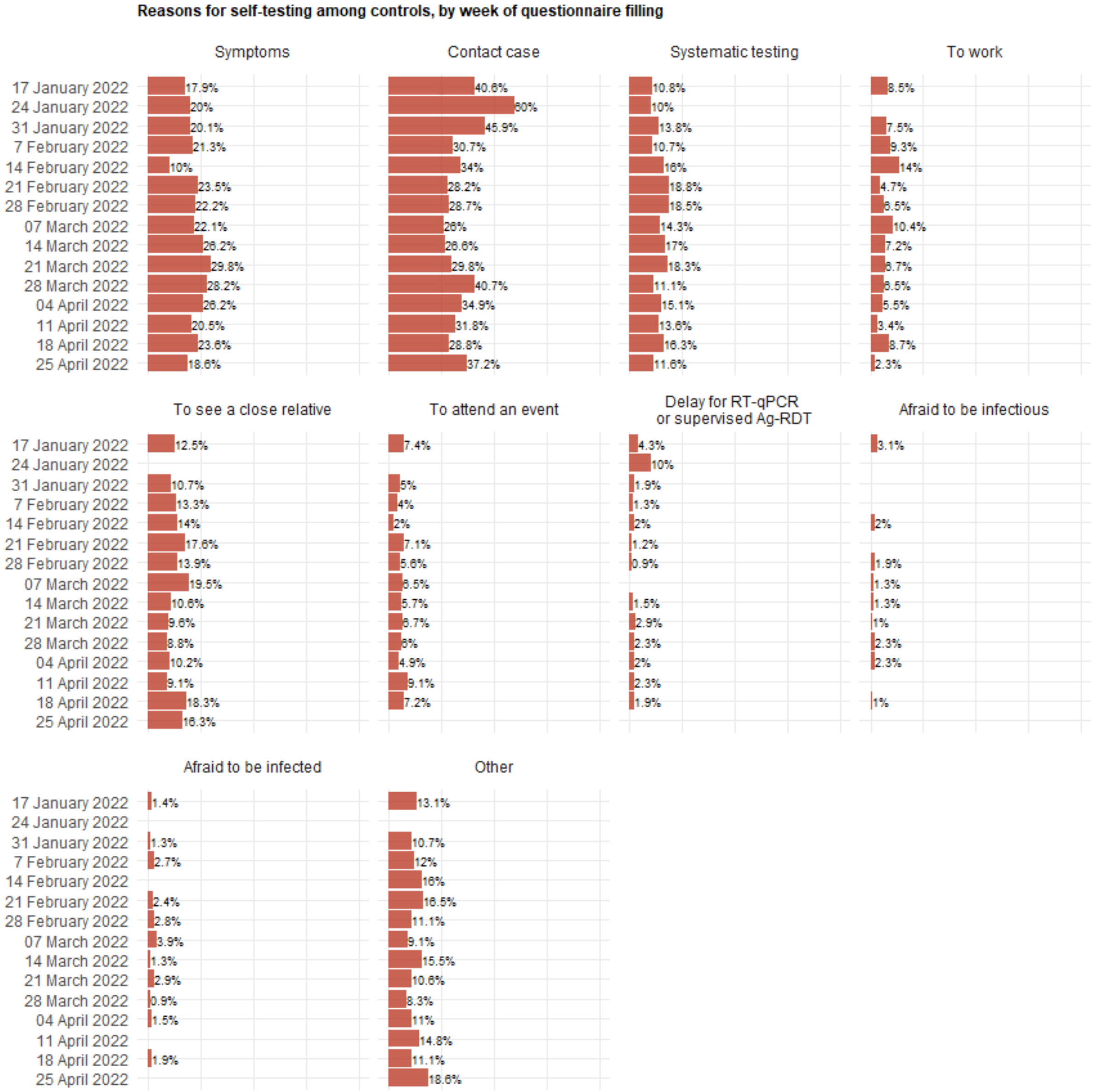

Supplementary Table S13: Comparison between French and study age pyramid by sex

Data for the French population as of January 2022 were retrieved from National Institute of Statistics and Economic Studies (Institut national de la statistique et des études économiques) website (https://www.insee.fr/fr/statistiques/2381474#figure1_radio2; accessed on 22 April 2023)

Age	French population in January 2022			Cases			Controls		
	Women	All	% women	Women	All	% women	Women	All	% women
[18-29]	4,198,851	8,479,026	50%	13,757	16,795	82%	1,185	1,855	64%
[30-39]	4,228,579	8,234,477	51%	33,241	41,292	81%	2,709	3,938	69%
[40-49]	4,353,072	8,561,785	51%	37,175	49,761	75%	3,316	5,297	63%
[50-59]	4,532,699	8,882,961	51%	26,143	37,773	69%	2,725	4,734	58%
[60-69]	4,248,966	8,061,417	53%	13,561	22,516	60%	2,149	4,051	53%
70+	6,022,556	10,354,663	58%	5,193	11,028	47%	951	2,319	41%
All	27,584,723	52,574,329	52%	129,070	179,165	72%	13,035	22,194	59%
